# Host Factor Nucleophosmin 1 (NPM1/B23) Exerts Antiviral Effects against Chikungunya Virus by Its Interaction with Viral Nonstructural Protein 3

**DOI:** 10.1128/spectrum.05371-22

**Published:** 2023-07-06

**Authors:** Parvanendhu Pradeep, Krishnankutty Chandrika Sivakumar, Easwaran Sreekumar

**Affiliations:** a Molecular Virology Laboratory, Rajiv Gandhi Centre for Biotechnology (RGCB), Thiruvananthapuram, India; b Research Centre, University of Kerala, Thiruvananthapuram, India; c Bioinformatics Facility, Rajiv Gandhi Centre for Biotechnology (RGCB), Thiruvananthapuram, India; d Molecular Bioassay Laboratory, Institute of Advanced Virology (IAV), Thiruvananthapuram, India; University of Manitoba

**Keywords:** CHIKV, alphavirus, NPM1, numatrin, NO38, viral macrodomain, nsP3, ADP-ribosylation

## Abstract

Chikungunya virus (CHIKV) hijacks host cell machinery to support its replication. Nucleophosmin 1 (NPM1/B23), a nucleolar phosphoprotein, is one of the host proteins known to restrict CHIKV infection; however, the mechanistic details of the antiviral role of NPM1 are not elucidated. It was seen in our experiments that the level of NPM1 expression affected the expression levels of interferon-stimulated genes (ISGs) that play antiviral roles in CHIKV infection, such as *IRF1*, *IRF7*, *OAS3*, and *IFIT1*, indicating that one of the antiviral mechanisms could be through modulation of interferon-mediated pathways. Our experiments also identified that for CHIKV restriction, NPM1 must move from the nucleus to the cytoplasm. A deletion of the nuclear export signal (NES), which confines NPM1 within the nucleus, abolishes its anti-CHIKV action. We observed that NPM1 binds CHIKV nonstructural protein 3 (nsP3) strongly via its macrodomain, thereby exerting a direct interaction with viral proteins to limit infection. Based on site-directed mutagenesis and coimmunoprecipitation studies, it was also observed that amino acid residues N24 and Y114 of the CHIKV nsP3 macrodomain, known to be involved in virus virulence, bind ADP-ribosylated NPM1 to inhibit infection. Overall, the results show a key role of NPM1 in CHIKV restriction and indicate it as a promising host target for developing antiviral strategies against CHIKV.

**IMPORTANCE** Chikungunya, a recently reemerged mosquito-borne infection caused by a positive-sense, single-stranded RNA virus, has caused explosive epidemics in tropical regions. Unlike the classical symptoms of acute fever and debilitating arthralgia, incidences of neurological complications and mortality were reported. Currently there are no antivirals or commercial vaccines available against chikungunya. Like all viruses, CHIKV uses host cellular machinery for establishment of infection and successful replication. To counter this, the host cell activates several restriction factors and innate immune response mediators. Understanding these host-virus interactions helps to develop host-targeted antivirals against the disease. Here, we report the antiviral role of the multifunctional host protein NPM1 against CHIKV. The significant inhibitory effect of this protein against CHIKV involves its increased expression and movement from its natural location within the nucleus to the cytoplasm. There, it interacts with functional domains of key viral proteins. Our results support ongoing efforts toward development of host-directed antivirals against CHIKV and other alphaviruses.

## INTRODUCTION

Chikungunya virus (CHIKV) is an *Aedes* sp. mosquito-transmitted alphavirus that belongs to the *Togaviridae* family and mainly causes acute fever, prolonged and debilitating arthralgia, and occasionally neurological complications in infants and elderly patients (1). Currently, there are no CHIKV-specific antivirals or vaccines available, and prevention of mosquito bites and usage of analgesics and anti-inflammatory drugs are still in practice (2). The development of effective therapies against CHIKV requires a better understanding of virus-host interactions, antiviral immunity, and the molecular mechanisms and cellular pathways used by the virus for its pathogenesis.

CHIKV is an enveloped virus with a positive-sense single-stranded RNA of about 11.8 kb that is capped at the 5′ end and polyadenylated at the 3′ end (3). CHIKV uses host cell machinery to replicate its genome. It has two open reading frames (ORFs), where the 5′ ORF encodes a nonstructural polyprotein (P1234) that is translated into four nonstructural proteins (nsPs) that form the RNA replicase complex, and the 3′ ORF encodes the structural proteins (C, E2, E1, 6K, and E3). The nonstructural proteins are involved in viral RNA replication, and the structural proteins help in viral assembly and budding of progeny virions. The nsP1234 polyprotein is cleaved into its subsequent components by the proteolytic activity of nsP2 and host proteases (4, 5). Of these, nsP1 is involved in RNA capping and membrane binding and has methyl and guanylyl transferase activities (6); nsP2, along with its protease activity, also has helicase and nucleoside triphosphatase (NTPase) activity (7); and nsP4 (RNA-dependent RNA polymerase) is a component of the replication complex that helps in viral genome replication (8).

The remaining nonstructural protein, nsP3, is a multifaceted viral protein that plays a role in ADP ribose hydrolysis, RNA binding, and host cell interactions and is essential for virus infection, pathogenesis, and adaptation to the host. The CHIKV nsP3 protein is composed of three domains, a highly conserved N-terminal macrodomain (MD); a central zinc-binding domain called an alpha virus unique domain (AUD), which is conserved among alphaviruses; and an unstructured, intrinsically disordered C-terminal hypervariable domain (HVD). The CHIKV nsP3 MD can hydrolyze ADP-ribose groups from mono-ADP-ribosylated (MARylated) proteins and is critical for CHIKV replication in vertebrate hosts, insect vectors, and mice (9, 10). The C-terminal HVD interacts with many cellular proteins and regulates viral replication and stress granule assembly (11, 12). During the CHIKV life cycle, nsP3 is localized in the viral replication complex (vRC) and acts as a platform for interaction with multiple host factors (13).

Several host proteins are involved in inhibiting virus replication and pathogenesis as part of cellular antiviral defense (14–16). Our previous study identified the antiviral role of the nuclear histone chaperone nucleophosmin-1 (NPM1/NO38/B23 or numatrin) in CHIKV infection (17, 18). This nucleolar phosphoprotein plays a role in cellular processes such as ribosome biogenesis, DNA replication and repair, stress response, centrosome duplication, and nucleo-cytoplasmic transport (19–22). NPM1 consists of an N-terminal oligomerization domain (OligoD), a central histone-binding domain (HBD), and a C-terminal RNA-binding domain (RBD) (23). NPM1 shuttles between the nucleus and the cytoplasm and has been implicated in infection with multiple viruses. NPM1 interacts with several viral protein components such as HIV-1 Rev (24, 25), Epstein Barr virus (EBV) EBNA2 (26), and human papillomavirus E6 and E7 (27).

In our previous study, we reported NPM1 aggregation in CHIKV-infected cells, and inhibition of this aggregation using an NPM1 oligomerization inhibitor (NSC348884) caused a significant increase in viral titer (18). In the present study, we further characterized the interaction between NPM1 and CHIKV, and identified the specific viral partner mediating this to interaction effect the antiviral activity and its mechanism.

## RESULTS

### Knockdown of NPM1 expression enhances CHIKV replication.

**FIG 1:**
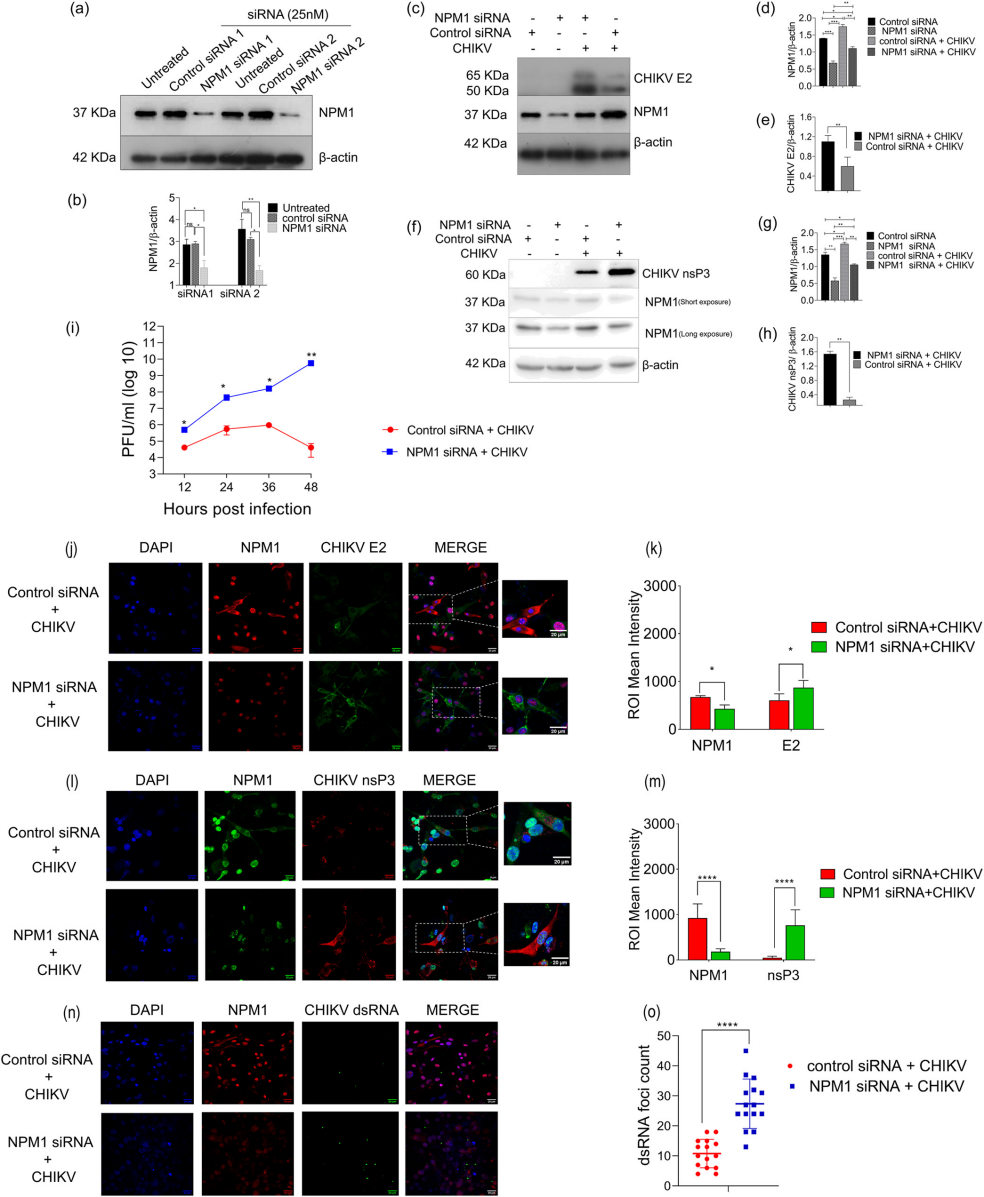
Knockdown of NPM1 expression enhances CHIKV replication. (a) Confirmation of siRNA efficiency. U-87 MG cells were transfected with 25 nM siRNA. NPM1 siRNA1 and NPM1 siRNA2 are two different siRNAs targeting NPM1. Control siRNA1 and control siRNA2 are nontargeting control pool siRNAs. Cells were harvested 24 h posttransfection, and the lysates were subjected to Western blotting. β-Actin was used as the loading control. (b) Densitometry analysis of NPM1 expression levels; *, *P* < 0.05; **, *P* < 0.005. Data were analyzed by two-way ANOVA and Sidak’s multiple-comparison test. (c) U-87 MG cells were transfected with respective siRNA1, and cells were mock infected or infected with CHIKV at an MOI of 1. Cells were harvested 48 h postinfection (p.i.), and lysates were subjected to Western blotting. Representative Western blot images for three independent experiments for NPM1, CHIKV E2 structural protein, and β-actin expression are shown. (d and e) Densitometry analysis of NPM1 (d) and CHIKV E2 (e) expression levels; **, *P* < 0.005; ***, *P* < 0.0005. Data were analyzed by ordinary one-way ANOVA. (f) Cell lysates were subjected to Western blotting for NPM1, CHIKV nsP3, and β-actin expression levels. (g and h) Densitometry analysis of NPM1 (g) and CHIKV nsP3 (h) expression levels; *, *P* < 0.05. Data were analyzed by one-way ANOVA. (i) Single-step virus growth assay. U-87 MG cells were transfected with NPM1 siRNA or control siRNA for 24 h, followed by CHIKV infection (MOI of 1), and the cell supernatant was collected from 12 h p.i. to 48 h p.i. for plaque assay; *, *P* < 0.05; **, *P* < 0.005. Data were analyzed by two-way ANOVA. (j) Immunofluorescence assays were performed on U-87 MG cells transfected with NPM1 siRNA1/control siRNA and infected with CHIKV (MOI of 1). Cells were stained for NPM1 and CHIKV envelope E2 expression at 48 h p.i.; scale bar, 20 μm. (k) Quantification of NPM1 and CHIKV E2 expression intensity; **, *P* < 0.005. Data were analyzed by two-way ANOVA and Sidak’s multiple-comparison test. (l) Immunofluorescence was performed on U-87 MG cells transfected with NPM1 siRNA1/control siRNA and infected with CHIKV (MOI of 1). Cells were stained for NPM1 and CHIKV nsP3 expression at 48 h p.i.; scale bar, 20 μm. (m) Quantification of NPM1 and CHIKV nsP3 expression intensity; **, *P* < 0.005. Data were analyzed by two-way ANOVA and Sidak’s multiple-comparison test. (n) Detection of dsRNA foci in control siRNA- or NPM1 siRNA1-treated infected cells; scale bar, 20 μm. (o) Quantification of dsRNA foci from 15 different fields from a total of three independent experiments (*n* = 3); ****, *P* < 0.0001. Data were analyzed by unpaired *t* test.

We used a short-interfering RNA (siRNA)-mediated approach to knock down the expression of NPM1 in U-87 MG cells using two different NPM1-targeted siRNAs (NPM1 siRNA1 and NPM1 siRNA2). Cells transfected with NPM1 siRNA1 or NPM1 siRNA2 expressed significantly less (1.5-fold) NPM1 protein (Fig. 1a and b) than cells transfected with negative-control siRNA. Expression of the CHIKV envelope protein E2 was markedly increased in NPM1-knockdown cells (NPM1-KD) compared with in control NPM1 cells infected with CHIKV (Fig. 1c to e). There was also a significant increase in the expression of nsP3 protein (Fig. 1f to h). At 36 h and 48 h postinfection (p.i.), viral yields in the NPM1-KD cells were 2-log_10_- and 5-log_10_-fold higher, respectively, than that observed in the control siRNA-transfected infected cells (Fig. 1i).

These observations were further substantiated by immunofluorescence results where we found that in NPM1-KD cells, there was increased expression of E2 protein compared to in control NPM1 cells after infection (Fig. 1j and k; Fig. S1a in the supplemental material). We also observed increased cytosolic granular expression of CHIKV nsP3 in NPM1-KD cells (Fig. 1l and m; Fig. S1b). Analysis of the production of double-stranded RNA (dsRNA) intermediates, a marker of viral replication complex assembly/genome replication intermediate, showed that at 48 h p.i., there is a massive increase in dsRNA-containing complexes in NPM1-KD cells compared to in NPM1 control cells after infection (Fig. 1n and o; Fig. S1c). Punctate cytoplasmic dsRNA foci staining was seen in infected cells, and the intensity of dsRNA staining, volume, or size of dsRNA foci and foci numbers were significantly increased in NPM1-KD infected cells.

### NPM1-mediated inhibition of CHIKV infection is pronounced at late time points of infection.

**FIG 2:**
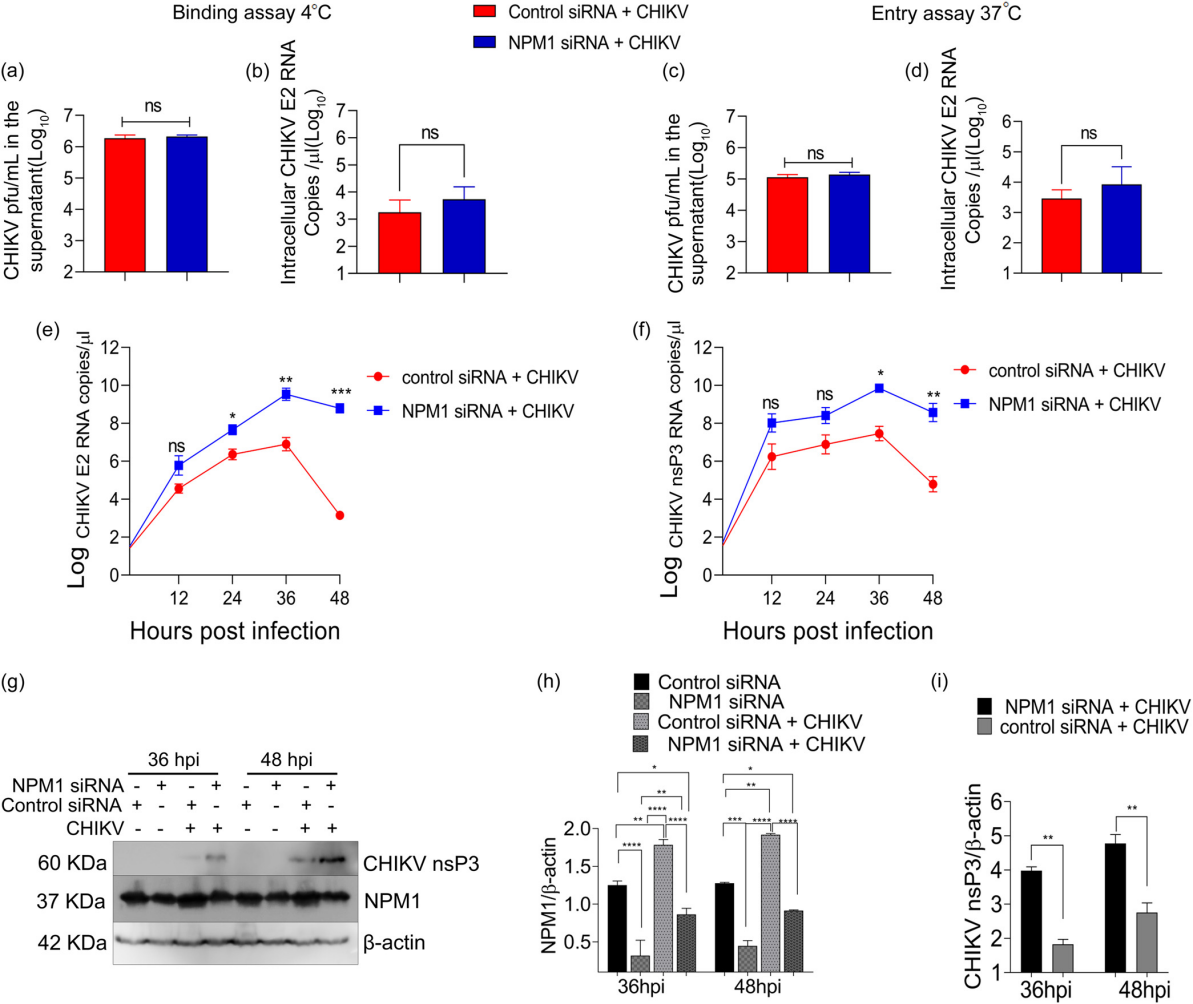
NPM1-mediated inhibition of CHIKV infection is pronounced at late time points of infection. (a to d) U-87 MG cells were transfected with control siRNA or NPM1 siRNA1 for 24 h. Cells were infected with CHIKV at an MOI of 1 at the indicated temperatures, and 1 h postadsorption, the culture supernatant for plaque assay and cell lysates after a PBS wash for RT-qPCR were collected; ns, not significant. (e and f) Total RNA was collected from cell lysates at the indicated time points, and CHIKV E2 and nsP3 intracellular viral RNA was quantified by RT-qPCR. CHIKV E2 and nsP3 RNA generated by *in vitro* transcription and quantified served as the template for generation of the standard curve; *, *P* < 0.05; **, *P* < 0.005; ***, *P* < 0.0005. Data were analyzed by two-way ANOVA. (g) U-87 MG cells were transfected with control or NPM1 siRNA1 and infected with CHIKV. Total protein lysates were collected at the indicated time points and analyzed by immunoblotting for CHIKV nsP3 and NPM1 with specific antibodies and β-actin as a loading control. Representative images of three independent experiments are shown. (h and i) Densitometry analysis of NPM1 (h) and CHIKV nsP3 (i) protein expression; *, *P* < 0.05; **, *P* < 0.005; ****, *P* < 0.0001. Data were analyzed by two-way ANOVA and Sidak’s multiple-comparison test.

We tested the role of NPM1 on viral attachment and entry as well as on the kinetics of viral replication (Fig. 2). There was no effect of NPM1 knockdown on viral binding or entry as revealed by no significant difference in unbound virus in the culture supernatant or intracellular viral RNA in control or NPM1-KD cells (Fig. 2a to d). Intracellular E2 RNA levels were compared at 0, 12, 24, 36, and 48 h p.i. in control and NPM1-KD cells. Between the NPM1-KD and control cells, the difference in viral E2 RNA levels at 24 h p.i. (*P* = 0.02) (Fig. 2e) was mild, but it was much more significant at 36 h and 48 h p.i. (*P* = 0.003 and 0.0003, respectively). Differences in CHIKV nsP3 RNA levels also followed a similar trend, although of a lesser magnitude (Fig. 2f). Viral nsP3 protein levels were also measured by Western blotting, revealing that at 36 and 48 h p.i., there was a significant increase in nsP3 levels in NPM1-KD cells compared to in NPM1 control cells (Fig. 2g to i).

### Overexpression of NPM1 suppresses CHIKV replication.

**FIG 3:**
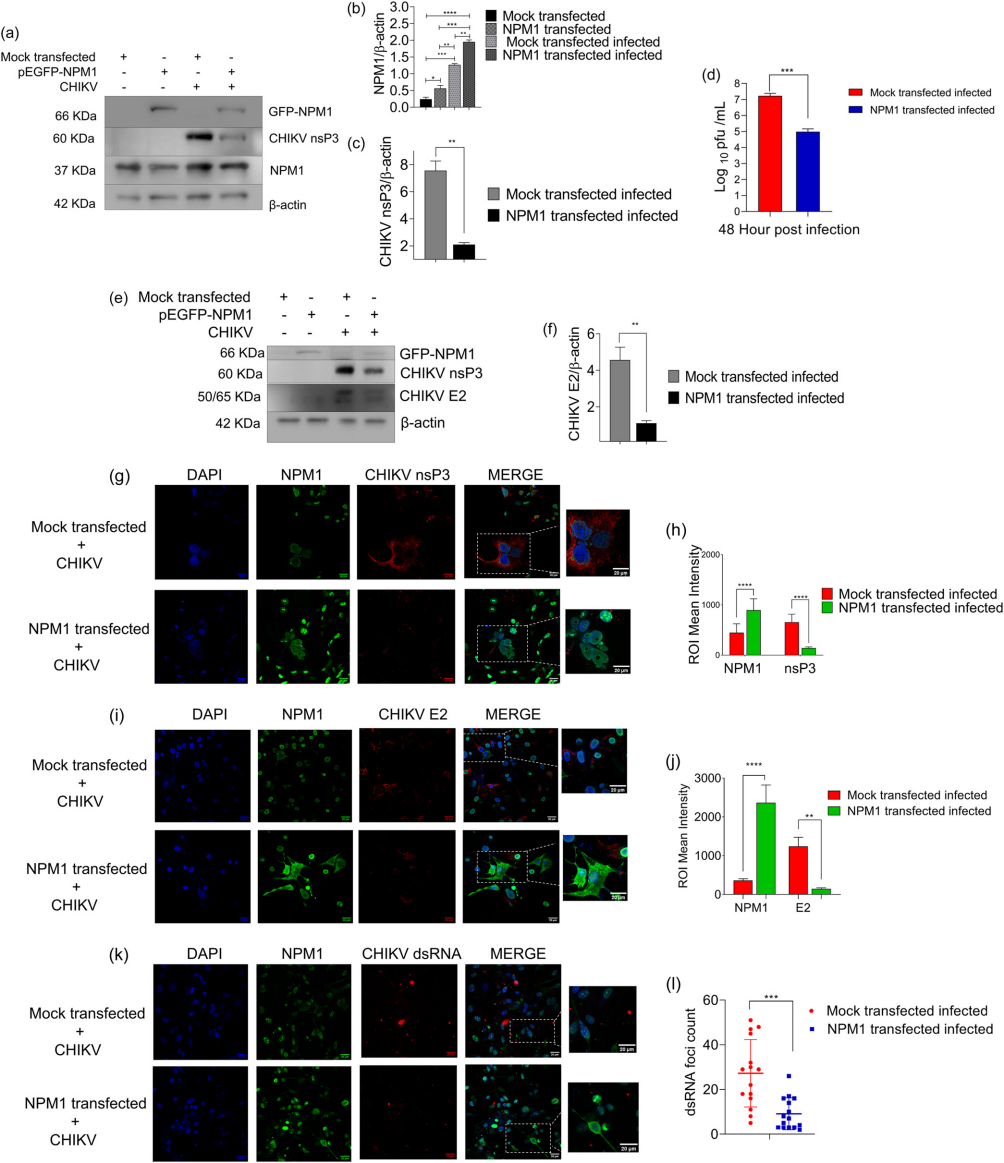
NPM1 overexpression suppresses CHIKV replication. (a) U-87 MG cells were transfected with mock vector or NPM1-expressing plasmid. Twenty-four hours posttransfection, cells were infected with CHIKV at an MOI of 1, and at 48 h p.i., cells were subjected to immunoblotting for endogenous NPM1, GFP-NPM1, and CHIKV nsP3 expression. β-Actin was used as a loading control. (b and c) Densitometry analysis of NPM1 (b) and CHIKV nsP3 (c); *, *P* < 0.05 (CHIKV nsP3); data were analyzed by unpaired *t* test; *, *P* < 0.05; **, *P* < 0.005; ****, *P* < 0.0001 (NPM1; data were analyzed by one-way ANOVA and Sidak’s multiple-comparison test). (d) Culture supernatants were harvested 48 h p.i., and viral titer was determined by plaque assay; ***, *P* < 0.0005. Data were analyzed by unpaired *t* test. (e) Lysates were subjected to immunoblotting for GFP-NPM1, CHIKV E2, and nsP3 expression. β-Actin was used as a loading control. (f) Densitometry analysis of CHIKV E2; *, *P* < 0.05. Data were analyzed by unpaired *t* test. (g) Immunofluorescence detection of NPM1 and CHIKV nsP3 expression. U-87 MG cells transfected with GFP-NPM1 vector or mock vector and infected with CHIKV (MOI of 1) 24 h posttransfection. Cells were fixed at 48 h p.i. and immunostained as indicated; scale bar, 20 μm. (h) Quantification of NPM1 and CHIKV nsP3 expression fluorescence intensity; ****, *P* < 0.001. Data were analyzed by two-way ANOVA and Sidak’s multiple-comparison test. (i) Immunofluorescence detection of NPM1 and CHIKV E2 expression. U-87 MG cells transfected with GFP-NPM1 vector or mock vector and infected with CHIKV (MOI of 1) 24 h posttransfection. Cells were fixed at 48 h p.i. and immunostained as indicated. (j) Quantification of NPM1 and CHIKV E2 expression fluorescence intensity; ****, *P* < 0.001. Data were analyzed by two-way ANOVA and Sidak’s multiple-comparison test. (k and l) The experiment was performed as in g, and cells were stained for dsRNA (k) and quantified (l). To quantify the dsRNA foci, we chose a total of 15 different fields from three independent experiments (*n* = 3); ***, *P* < 0.0005. Data were analyzed by unpaired *t* test; scale bar, 20 μm).

A significant increase in NPM1 levels was observed 24 h posttransfection in U-87 MG cells after transfection with NPM1-expressing plasmid (Fig. 3a). In these cells, infection with CHIKV at a multiplicity of infection (MOI) of 1 led to reduced CHIKV nsP3 protein expression (Fig. 3a to c) and an approximately 2-log_10_ reduction in viral titer compared to mock vector-transfected infected cells at 48 h p.i. (Fig. 3d). There was also a significant reduction in CHIKV E2 protein expression (Fig. 3e and f). Immunofluorescence analysis showed that after NPM1 overexpression, there was a significant reduction in cytosolic granular expression of nsP3 compared to that observed in mock-transfected infected cells (Fig. 3g and h; Fig. S2a). We also observed a reduction in the expression of CHIKV E2 in NPM1-transfected cells (Fig. 3i and j; Fig. S2b) and a significant reduction in the number of dsRNA-containing complexes at 48 h p.i. in NPM1-transfected cells (Fig. 3k and l; Fig. S2c).

### NPM1 is required for interferon (IFN)-stimulated gene (ISG) expression in response to CHIKV infection.

**FIG 4:**
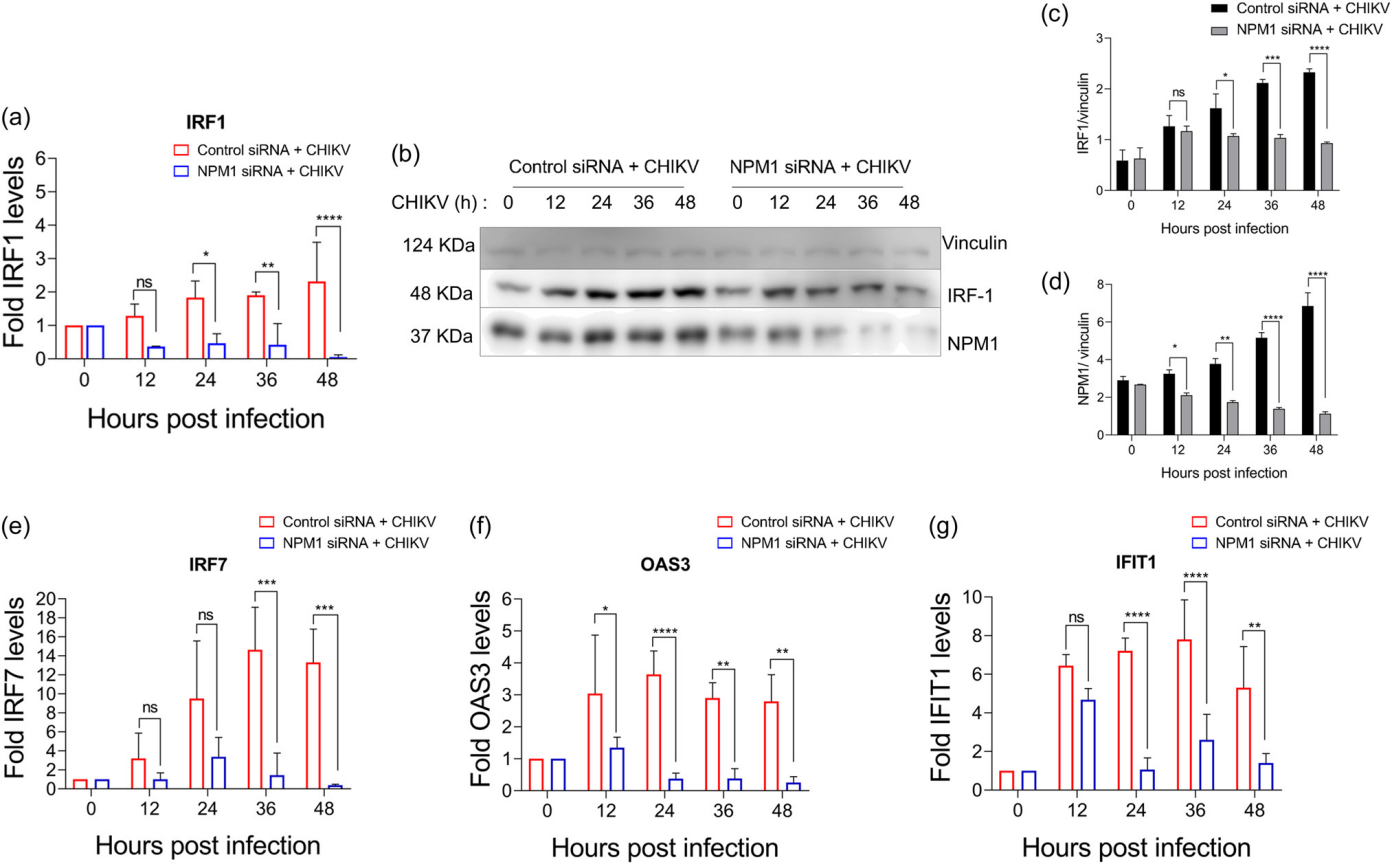
NPM1 is required for interferon-stimulated gene (ISG) expression in response to CHIKV infection. (a) U-87 MG cells were transfected with control siRNA or NPM1 siRNA2 for 24 h. Cells were infected with CHIKV at an MOI of 1, and total RNA was collected from cell lysates at 0, 12, 24, 36, and 48 h p.i.; *IRF1* transcript levels were quantified by RT-qPCR. Data were normalized by the level of *ACTB* expression in each sample. The 2^−ΔΔ^*^CT^* method was used to calculate relative expression changes. Data were collected and are expressed as mean ± standard deviation (SD) of three independent experiments; *, *P* < 0.05; **, *P* < 0.005; ***, *P* < 0.0005. Data were analyzed by two-way ANOVA. (b) Total protein lysates were collected at the indicated time points and analyzed by immunoblotting for IRF-1 and NPM1 with specific antibodies and vinculin as a loading control. Representative images of three independent experiments are shown. (c and d) Densitometry analysis of IRF-1 (c) and NPM1 (d) protein expression; *, *P* < 0.05; **, *P* < 0.005; ***, *P* = 0.0001; ****, *P* < 0.0001. Data were analyzed by two-way ANOVA and Sidak’s multiple-comparison test. (e to g) Experiments were performed as in a, and *IRF7* (e), *OAS3* (f), and *IFIT1* (g) transcripts were quantified by RT-qPCR. Data were normalized by the level of *ACTB* expression in each sample. The 2^−ΔΔ^*^CT^* method was used to calculate relative expression changes. Data were collected, and the mean ± SD of three independent experiments are shown; *, *P* < 0.05; **, *P* < 0.005; ***, *P* < 0.0005. Data were analyzed by two-way ANOVA.

To investigate the role of NPM1 on ISG induction, we examined the effect of NPM1 depletion on the expression of ISGs such as *IRF1*, *IRF7*, *OAS3*, and *IFIT1* using real-time quantitative PCR (RT-qPCR) and Western blotting. *IRF1* transcript levels were compared at 0, 12, 24, 36, and 48 h p.i. in control and NPM1-KD-infected cells (Fig. 4a). The RT-qPCR data showed that *IRF1* levels in NPM1-KD cells were 1.3-fold, 1.4-fold, and 2.2-fold lower than those observed in control cells at 24, 36, and 48 h p.i., respectively (*P* < 0.01, *P* < 0.0055, and *P* < 0.0001, respectively). We also observed a significant reduction in IRF-1 protein levels after NPM1 silencing in infected cells in a time-dependent manner (Fig. 4b to d). At the transcript level, *IRF7* levels were 13-fold and 12-fold lower in NPM1-KD cells at 36 and 48 h p.i., respectively (*P* < 0.0001) (Fig. 4e).We also found that *OAS3* levels were 1.6-fold, 3.2-fold, 2.5-fold, and 2.5-fold lower in NPM1-KD cells at 12, 24, 36, and 48 h p.i., respectively (*P* = 0.04, *P* = 0.0001, *P* = 0.0016, and *P* = 0.0015, respectively) (Fig. 4f), whereas *IFIT1* levels were downregulated 6.1-fold, 5.1-fold, and 3.9-fold at 24, 36, and 48 h p.i., respectively (*P* < 0.0001, *P* < 0.0001, and *P* = 0.0017, respectively) (Fig. 4g), indicating that depletion of NPM1 affects the levels of ISGs induced by CHIKV infection.

### NPM1 overexpression upregulates ISG expression.

**FIG 5:**
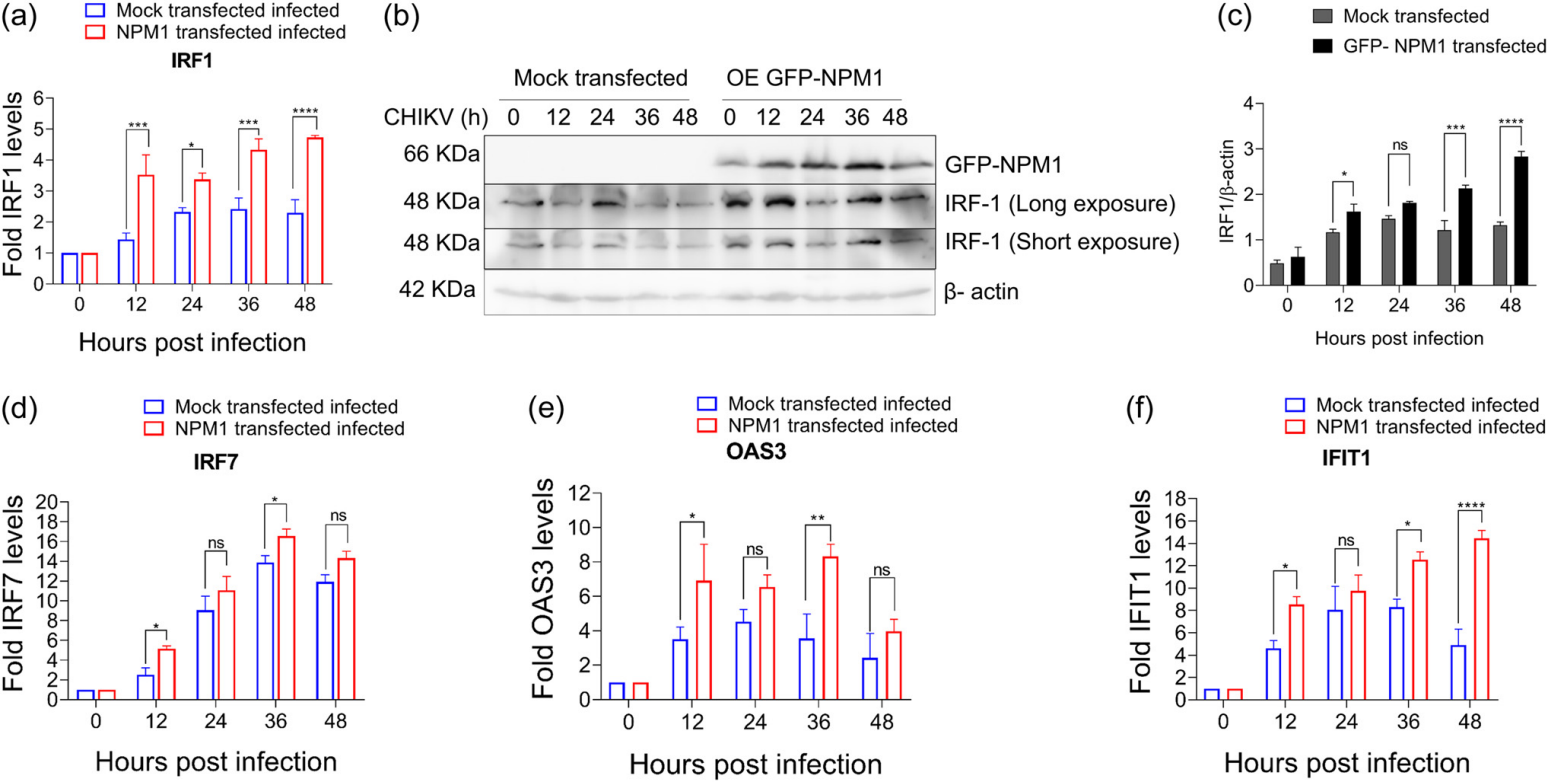
NPM1 overexpression upregulates ISG expression. (a) U-87 MG cells were mock transfected or GFP-NPM1 transfected, and 24 h posttransfection, cells were infected with CHIKV at an MOI of 1. Total RNA was collected from cell lysates at 0, 12, 24, 36, and 48 h p.i., and *IRF1* transcript levels were quantified by RT-qPCR. Data were normalized by the level of *ACTB* expression in each sample. The 2^−ΔΔ^*^CT^* method was used to calculate relative expression changes. Data were collected and expressed as mean ± SD of three independent experiments; *, *P* < 0.05; **, *P* < 0.005; ***, *P* < 0.0005. Data were analyzed by two-way ANOVA. (b) Total protein lysates were collected at the indicated time points and analyzed by immunoblotting for IRF-1 and GFP-NPM1 with specific antibodies and β-actin as a loading control. Representative images of three independent experiments are shown. (c) Densitometry analysis of IRF-1 protein expression; *, *P* < 0.05; **, *P* < 0.005; ***, *P* = 0.0001; ****, *P* < 0.0001. Data were analyzed by two-way ANOVA and Sidak’s multiple-comparison test. (d to f) Experiments were performed as in a, and *IRF7* (d), *OAS3* (e), and *IFIT1* (f) transcripts were quantified by RT-qPCR. Data were normalized by the level of *ACTB* expression in each sample. The 2^−ΔΔ^*^CT^* method was used to calculate relative expression changes. Data were collected and are expressed as mean ± SD of three independent experiments; *, *P* < 0.05; **, *P* < 0.005; ***, *P* < 0.0005. Data were analyzed by two-way ANOVA.

We also investigated the role of NPM1 in the regulation of these ISGs by examining the effects of NPM1 overexpression on the expression of ISGs such as *IRF1*, *IRF7*, *OAS3*, and *IFIT1* using RT-qPCR and Western blotting. *IRF1* transcript levels were compared at 0, 12, 24, 36, and 48 h p.i. in mock-transfected infected cells and in infected cells transfected with green fluorescent protein (GFP)-labeled NPM1 (GFP-NPM1) (Fig. 5a). RT-qPCR data showed that *IRF1* levels in GFP-NPM1-transfected cells were 2-fold, 1-fold, 1.9-fold, and 2.4-fold higher than those observed in mock-transfected infected cells at 12, 24, 36, and 48 h p.i., respectively (*P* = 0.0002, *P* = 0.0337, *P* = 0.0005, and *P* < 0.0001, respectively). We also observed a significant increase in the expression of IRF-1 protein after NPM1 overexpression in infected cells in a time-dependent manner (Fig. 5b and c). At the transcript level, *IRF7* levels were 2.63-fold and 2.68-fold higher in GFP-NPM1-overexpressing cells at 12 and 36 h p.i., respectively (*P* < 0.05) (Fig. 5d).We also found that *OAS3* levels were 3.4-fold and 4.7-fold higher in GFP-NPM1-overexpressing cells at 12 and 36 h p.i., respectively (*P* < 0.05 and *P* < 0.005, respectively) (Fig. 5e), whereas *IFIT1* levels were upregulated 3.9-fold, 1.7-fold, 4.2-fold, and 9.5-fold at 12, 24, 36, and 48 h p.i., respectively (*P* = 0.01, *P* = 0.5, *P* < 0.01, and *P* = 0.0001) (Fig. 5f), indicating that overexpression of NPM1 upregulates the levels of ISGs induced by CHIKV infection.

### NPM1 is translocated to the cytoplasm during CHIKV infection.

**FIG 6:**
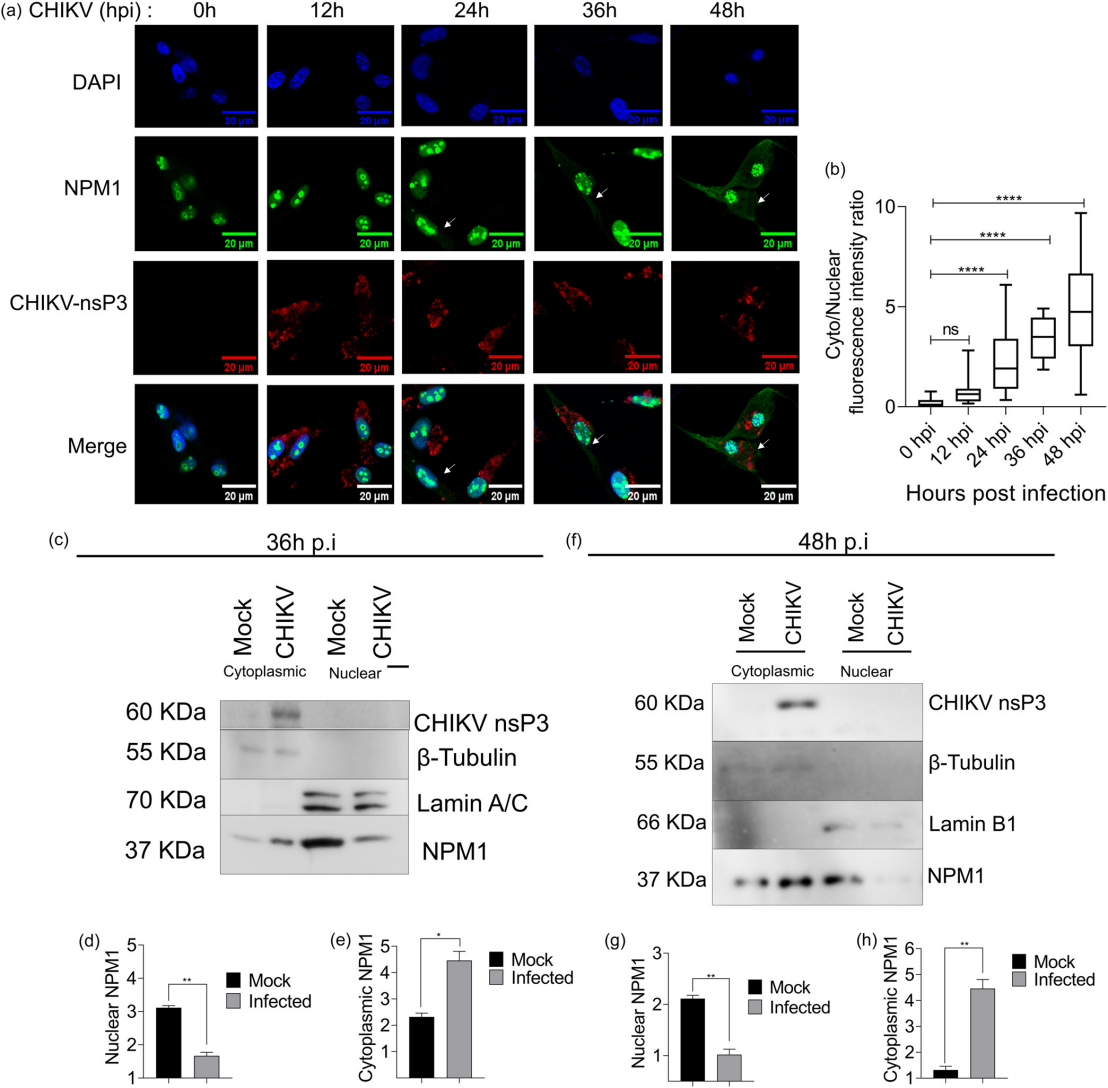
NPM1 is translocated to the cytoplasm during infection. (a) U-87 MG cells were mock infected or infected with CHIKV (MOI of 1), fixed at 0, 12, 24, 36, and 48 h p.i., and immunostained for NPM1 and CHIKV nsP3. Arrows indicate the cytoplasmic translocation of NPM1; scale bar, 20 μm. (b) Quantification of the cytoplasmic:nuclear fluorescence intensity ratio represented as a box plot; *n* = 21 cells from three biological replicates. Whiskers represent 1st to 99th percentiles; ****, *P* < 0.0001. Data were analyzed by ordinary one-way ANOVA. (c) Proteins derived from nuclear and cytoplasmic fractions at 36 h p.i. were subjected to Western blotting. Samples were probed for CHIKV nsP3 and NPM1, with β-tubulin and lamin A/C as loading controls for cytoplasmic fractions and nuclear fractions, respectively. (d and e) Densitometry analysis of nuclear NPM1 (d) and cytoplasmic NPM1 (e) levels; *, *P* < 0.05; **, *P* < 0.005. Data were analyzed by unpaired *t* test. (f) Cell lysates from nuclear and cytoplasmic fractions at 48 h p.i. were subjected to Western blotting. Samples were probed for CHIKV nsP3 and NPM1, with β-tubulin and lamin B1 as loading controls for cytoplasmic fractions and nuclear fractions, respectively. (g and h) Densitometry analysis of nuclear NPM1 (g) and cytoplasmic NPM1 (h) levels; *, *P* < 0.05; **, *P* < 0.005. Data were analyzed by unpaired *t* test.

Viral infections can modulate the expression of intracellular proteins or cause their relocalization as an innate response against infection (28). To determine whether the localization of NPM1 changes during infection, we analyzed the kinetics of NPM1 localization during infection and analyzed the levels of NPM1 in nuclear and cytoplasmic compartments. While in control cells most NPM1 resided in the nucleus (as expected), after CHIKV infection until 12 h p.i., NPM1 was predominantly present in the nuclear region, and there was a slight translocation of NPM1 from the nucleus to the cytoplasm at 24 h p.i., and at 36 h p.i. onward, there was slight reduction in expression of nuclear NPM1 and a significant increase in the levels of cytoplasmic NPM1 (Fig. 6a and b). We also performed a subcellular fractionation assay of mock- and CHIKV-infected cells at 36 h p.i. (Fig. 6c to e) and 48 h p.i. (Fig. 6f to h) followed by immunoblotting and observed an increase in cytoplasmic NPM1 aggregation in infected cells.

### Cytoplasmic relocalization of NPM1 is important for its anti-CHIKV activity.

**FIG 7:**
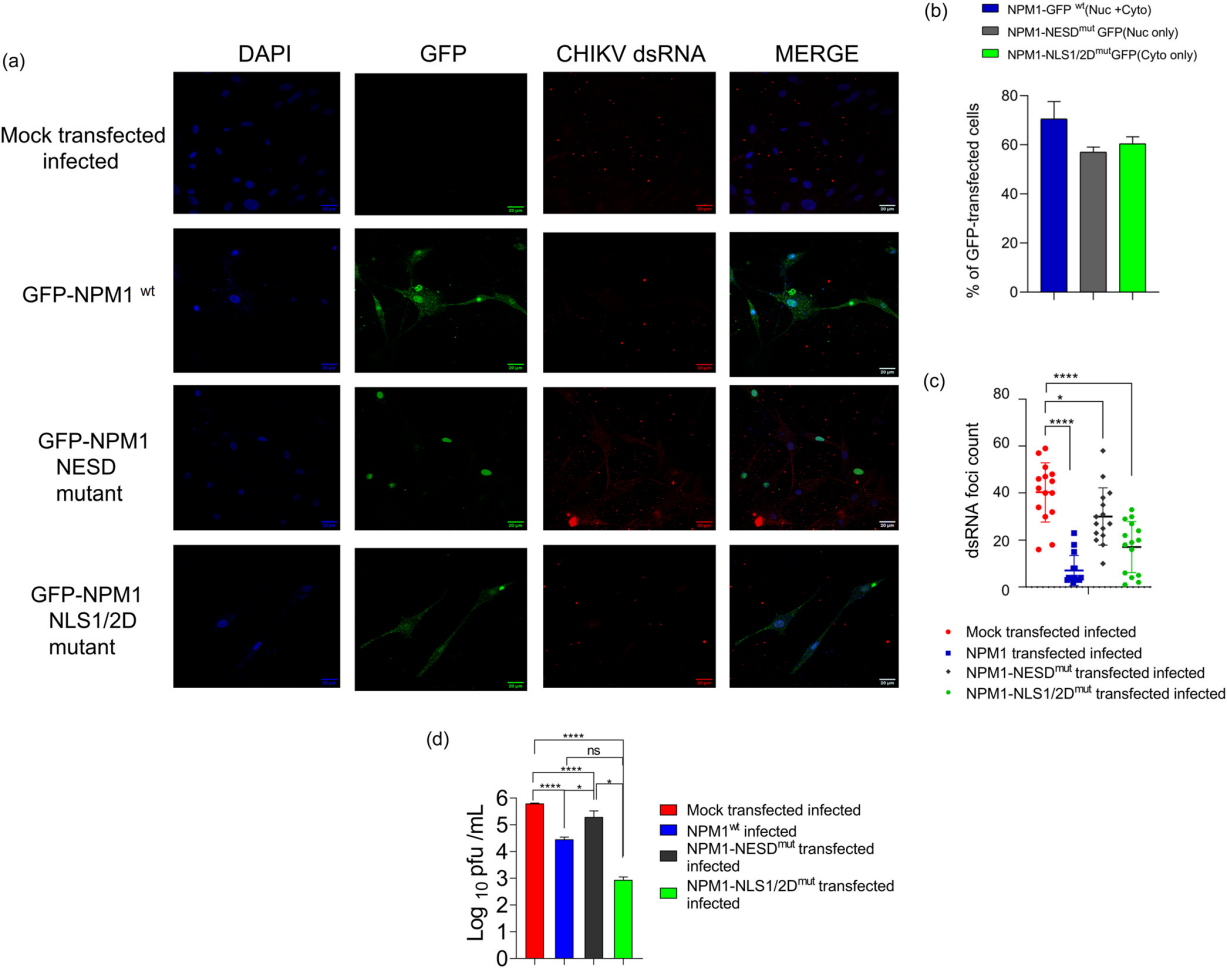
Cytoplasmic relocalization of NPM1 is important for its anti-CHIKV activity. (a) U-87 MG cells were transfected with mock vectors, NPM1 expression vectors containing a wild-type construct, an NPM1-NESD mutant that has a nuclear export sequence deletion, or an NPM1-NLS1/2D mutant that has a nuclear localization signal deletion. Twenty-four hours after transfection, cells were infected with CHIKV at an MOI of 1, and cells were fixed at 48 h p.i., stained with an antibody to GFP and anti-dsRNA, and imaged by confocal microscopy; ****, *P* < 0.0001; **, *P* < 0.005. Data were analyzed by ordinary one-way ANOVA and Sidak’s multiple-comparison test; scale bar, 20 μm. (b) Cells were scored, and the percentage of wild-type GFP-NPM1 and mutant construct-transfected and infected cells are represented; ****, *P* < 0.0001. Data were analyzed by ordinary one-way ANOVA and Sidak’s multiple-comparison test. (c) Quantification of dsRNA foci, a marker of viral genome replication, in mock plasmid-, NPM1 wild-type (NPM1wt)-, NPM1-NESD-, and NPM1-NLS1/2D plasmid-transfected infected cells at 48 h p.i. We chose cells that are both infected and expressing transfected GFP-NPM WT or mutated constructs from 15 different fields from three independent experiments. (d) Experiments were done as in a, and the cell culture supernatants at 48 h p.i. were subjected to plaque assay to determine virus yield.

NPM1 protein has a nuclear localization signal (NLS) in its histone-binding central domain and a nuclear export signal (NES) motif in its N terminus, which also has a functional oligomerization motif. To investigate the importance of NPM1 relocalization on CHIKV replication, we transfected wild-type NPM1 (NPM1wt), NPM1 mutant construct (NPM1-NESD) that has a deletion of the NES, and an NLS-deleted construct NPM-NLS1/2D followed by CHIKV infection (MOI of 1) 24 h posttransfection. When the NPM1-NESD construct is expressed, it remains in the nucleus and is no longer expressed in the cytoplasm, whereas NPM-NLS1/2D is expressed only in the cytoplasm (29). We checked dsRNA foci and observed a significant increase in the number and volume/size of dsRNA foci in NPM1-NESD-transfected infected cells compared to in NPM1wt-transfected cells at 48 h p.i., whereas we observed a significant reduction in the number of dsRNA foci in NPM1-NLS1/2D-transfected cells (Fig. 7a and c; Fig. S3). It was found that there was an increase in viral titer (1-log_10_, *P* = 0.01) in NPM1-NESD-expressing cells compared to in NPM1wt-transfected cells after infection, whereas NPM1-NLS1/2D-expressing cells had a reduction in viral titer (Fig. 7d).

### NPM1 binds CHIKV nsP3.

**FIG 8:**
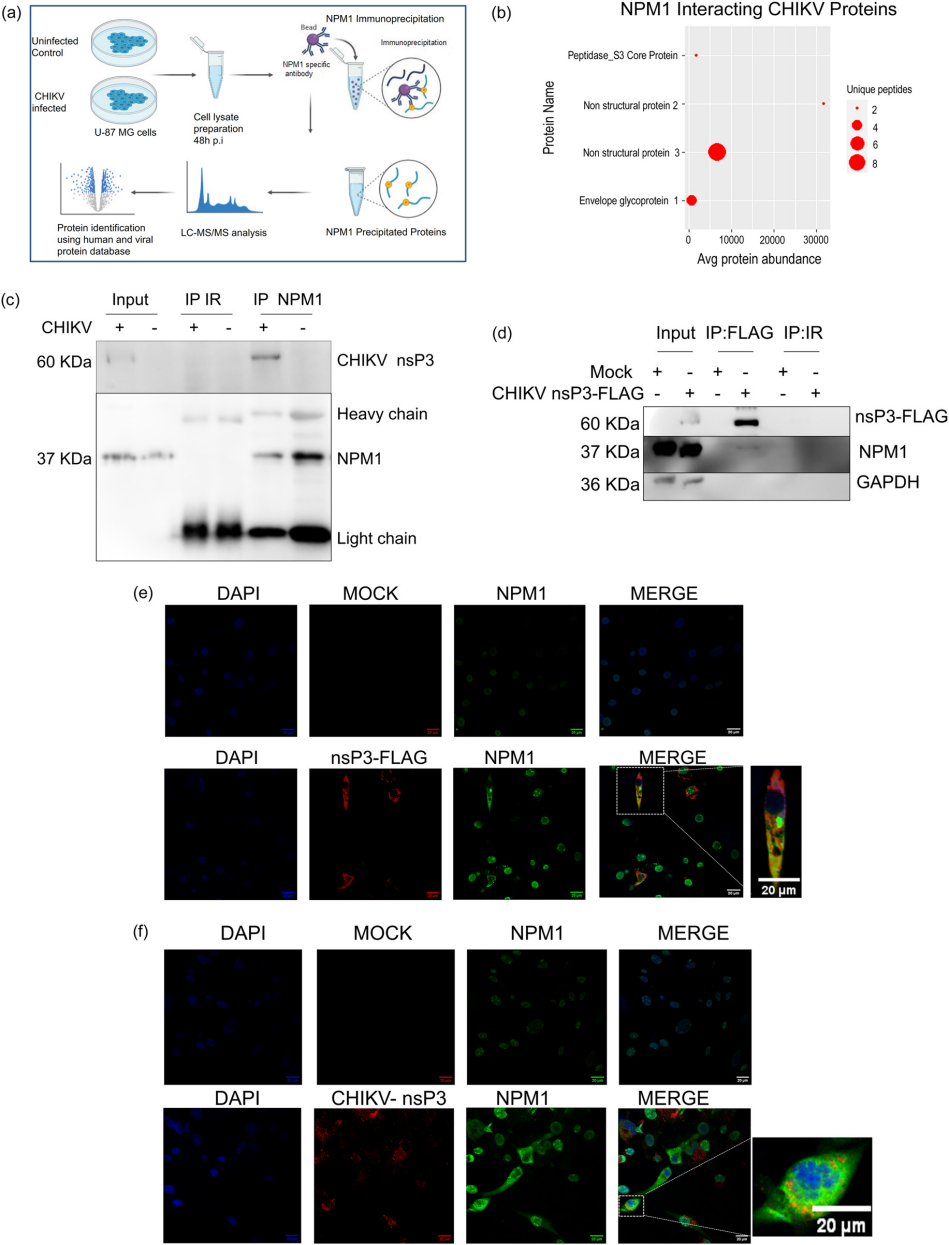
NPM1 predominantly binds to CHIKV nsP3. (a) Scheme of coimmunoprecipitation and LC-MS/MS analysis. (b) Bubble plot that represents identified NPM1 binding proteins in the viral protein database. (c) U-87 MG cells were mock infected or CHIKV infected at an MOI of 1; cell lysates were prepared 48 h postinfection and immunoprecipitated with NPM1 antibody or nonspecific IgG (IR). Protein samples were separated by SDS-PAGE and detected with antibodies against FLAG-nsP3 and NPM1 by Western blotting. (d) Cells were transfected with CHIKV nsP3-FLAG construct, and whole-cell extracts were prepared for coimmunoprecipitation assays using anti-FLAG antibody or nonspecific IgG (IR). Protein complexes were separated by SDS-PAGE and detected by Western blotting with antibodies against NPM1, GAPDH, and FLAG. Data are representative of three independent experiments. (e) Colocalization of NPM1 with FLAG-nsP3. U-87 MG cells were mock transfected or CHIKV nsP3-FLAG transfected, fixed at 48 h posttransfection, and stained for expression of NPM1 and nsP3-FLAG. Representative images of three independent experiments are shown; scale bar, 20 μm. The highlighted region indicates colocalization. (f) Colocalization of NPM1 with CHIKV nsP3. U-87 MG cells were mock or CHIKV infected, fixed at 48 h p.i., and stained for expression of NPM1 and CHIKV nsP3.

NPM1 coimmunoprecipitation and liquid chromatography-tandem mass spectrometry (LC-MS/MS) analyses were performed as per the scheme depicted in Fig. 8a to identify viral proteins that bind NPM1. Based on the maximum number of unique peptides identified, it was observed that NPM1 predominantly binds CHIKV nsP3 and weakly interacts with CHIKV E1 protein (Fig. 8b). Western blotting of the lysate from infected cells revealed that CHIKV nsP3 coprecipitated with NPM1 (Fig. 8c). In a reciprocal or reverse coimmunoprecipitation assay using anti-FLAG antibody in HEK293T cells transfected with CHIKV nsP3 expressing FLAG, we could detect NPM1 in the immunoprecipitated complex (Fig. 8d). Further, immunofluorescence staining of U-87 MG cells transfected with nsP3-FLAG showed colocalization of NPM1 with nsP3, predominantly in perinuclear and cytoplasmic regions (Pearson correlation coefficient of 0.745) (Fig. 8e). In CHIKV-infected cells, immunofluorescence staining showed colocalization of NPM1 with CHIKV nsP3 with a Pearson’s correlation coefficient (PCC) of 0.6 (Fig. 8f).

### NPM1 strongly binds the macrodomain of CHIKV nsP3.

**FIG 9:**
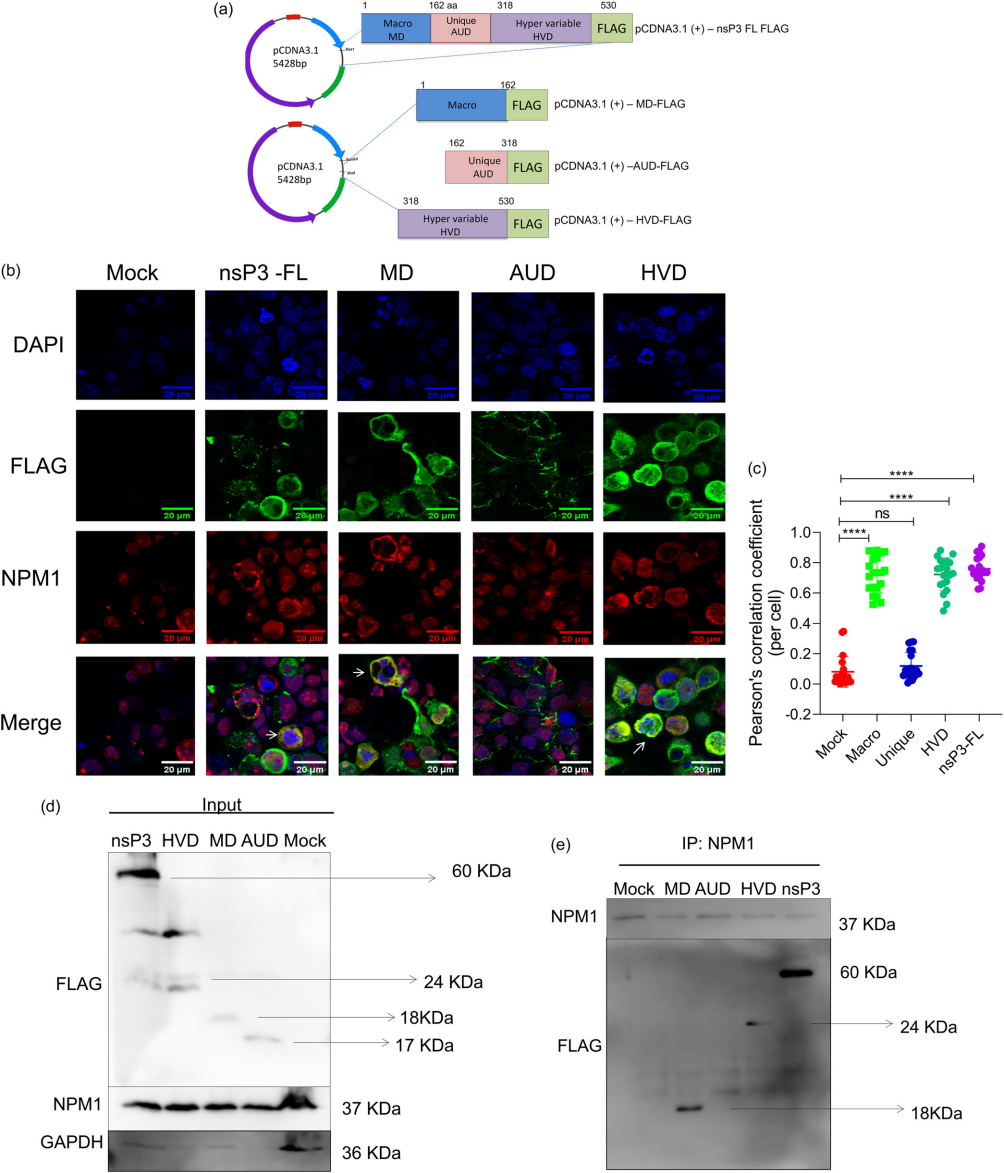
NPM1 strongly binds with the macrodomain of CHIKV nsP3. (a) Schematic representation of CHIKV nsP3-FLAG and its truncated derivatives. (b) HEK293T cells were transfected with CHIKV nsP3-FLAG full-length plasmid, macro (MD-FLAG), unique (AUD-FLAG), and hypervariable domain (HVD-FLAG) truncated constructs and costained with NPM1 and FLAG antibodies. Nuclei were stained with DAPI, and cells were imaged by confocal microscopy; scale bar, 20 μm. (c) Pearson correlation coefficients of colocalization of NPM1 with respective nsP3 subdomains were assessed using Fiji image processing software. White arrows indicate a colocalized region. Scatter dot plot with Pearson correlation coefficients and mean with SD were calculated from 20 selected cells in each group; ****, *P* < 0.0001; **, *P* < 0.005; *, *P* < 0.05. Data were analyzed by ordinary one-way ANOVA. (d and e) Total cell extracts were prepared for each condition and coimmunoprecipitated with an anti-NPM1 antibody. Proteins and bound complexes were immunoblotted for FLAG, NPM1, or GAPDH as indicated. Molecular masses are indicated in the blot. Results are representative of three independent experiments.

Plasmids encoding the nsP3 N-terminal macrodomain (MD), the central zinc-binding unique domain (AUD), or C-terminal hypervariable domain (HVD) with a C-terminal FLAG tag were generated (Fig. 9a). In plasmid-transfected cells, while full-length nsP3 was predominantly observed in the perinuclear region and cytoplasm, the MD and HVD proteins were found to be uniformly distributed in the nucleus and cytoplasm (Fig. 9b). By contrast, AUD was detected as helical or filament-like structures outside the nuclear membrane. Anti-NPM1 antibody staining revealed that NPM1 is predominantly present in the nucleus and colocalizes with cells expressing MD, HVD, and nsP3 full-length proteins (Fig. 9b and c). In NPM1-binding affinity assays for each subdomain of nsP3, full-length nsP3 protein and MD reproducibly immunoprecipitated with anti-NPM1 antibody under high-stringency wash conditions. However, under less stringent wash conditions with 0.25% Triton X-100 and 0.1% NP-40, HVD-FLAG also bound NPM1 (Fig. 9d and e).

### NPM1 is a target substrate for ADP ribosylation.

**FIG 10:**
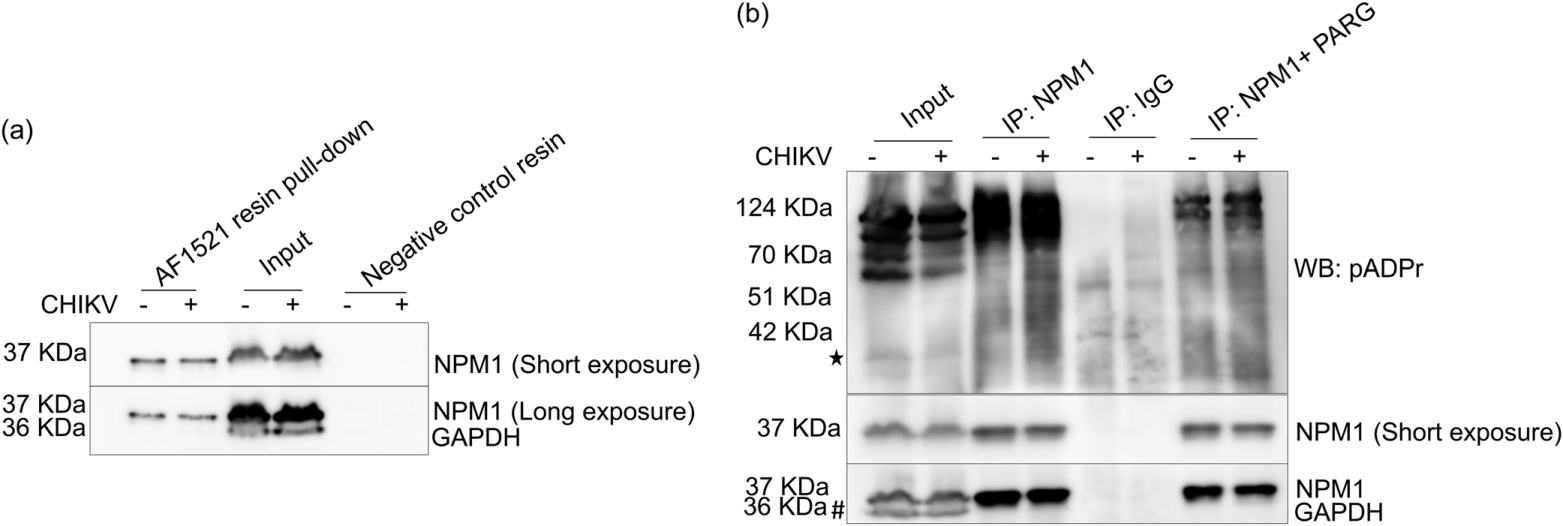
NPM1 is a target substrate for ADP ribosylation. (a) Macrodomain pulldown assay. U-87 MG cells were either mock infected or CHIKV infected at an MOI of 1, and lysates were used for pulldown using AF1521 macrodomain covalent resin and negative-control resin. The bound proteins were eluted and detected by Western blotting with an NPM1 antibody. The image is representative of three independent experiments. (b) U-87 MG cells were either mock infected or CHIKV infected at an MOI of 1, and lysates were treated with or without PARG (100 ng), immunoprecipitated using NPM1 antibody, and blotted with anti-pan-ADP-ribose binding reagent (pADPr).The blot is overexposed to facilitate the detection of less-abundant PARylated proteins. Asterisks indicate the position of the corresponding NPM1 protein. The “#” symbol indicates the band corresponding to GAPDH protein levels. Levels of NPM1 protein expression in the lysates are also shown.

To determine whether NPM1 is ADP ribosylated, we used AF1521 macrodomain covalent resin and negative-control covalent resin to pull down mono-ADP-ribosylated (MARylated) and poly-ADP-ribosylated (PARylated) proteins from control and CHIKV-infected cell lysates. Resin-bound proteins were eluted and subjected to immunoblotting using NPM1 antibody. Western blotting data showed that NPM1 immunoprecipitated with the AF1521 macrodomain covalent resin, whereas negative-control resin showed no pulldown (Fig. 10a). To further understand the ADP ribosylation status of NPM1 in control and CHIKV-infected cells, lysates were treated with poly-ADP-ribose glycohydrolase (PARG), which converts poly-ADP-ribose chains into mono-ADP-ribose chains. Samples were subjected to immunoprecipitation using NPM1 antibody. Immunoprecipitated proteins were probed with anti-pan-ADP-ribose binding agent that selectively binds both MARylated and PARylated proteins. Immunoblotting data showed a specific signal corresponding to NPM1 in the immunoprecipitated lanes under both conditions. This indicated that NPM1 is a target substrate for mono- and poly-ADP ribosylation. Because of the uneven addition of ADP-ribose units to individual NPM1 molecules, the signals form smears rather than a discrete band (Fig. 10b). In the anti-poly-ADP-ribose blot, preincubation of the lysate with PARG was found to decrease total poly-ADP ribosylation levels in the immunoprecipitated proteins, confirming that the signals are indeed due to poly-ADP ribosylation.

### *In silico* analysis predicts that NPM1 interaction with the CHIKV nsP3 macrodomain is mediated by ADP-ribose-binding residues.

**FIG 11:**
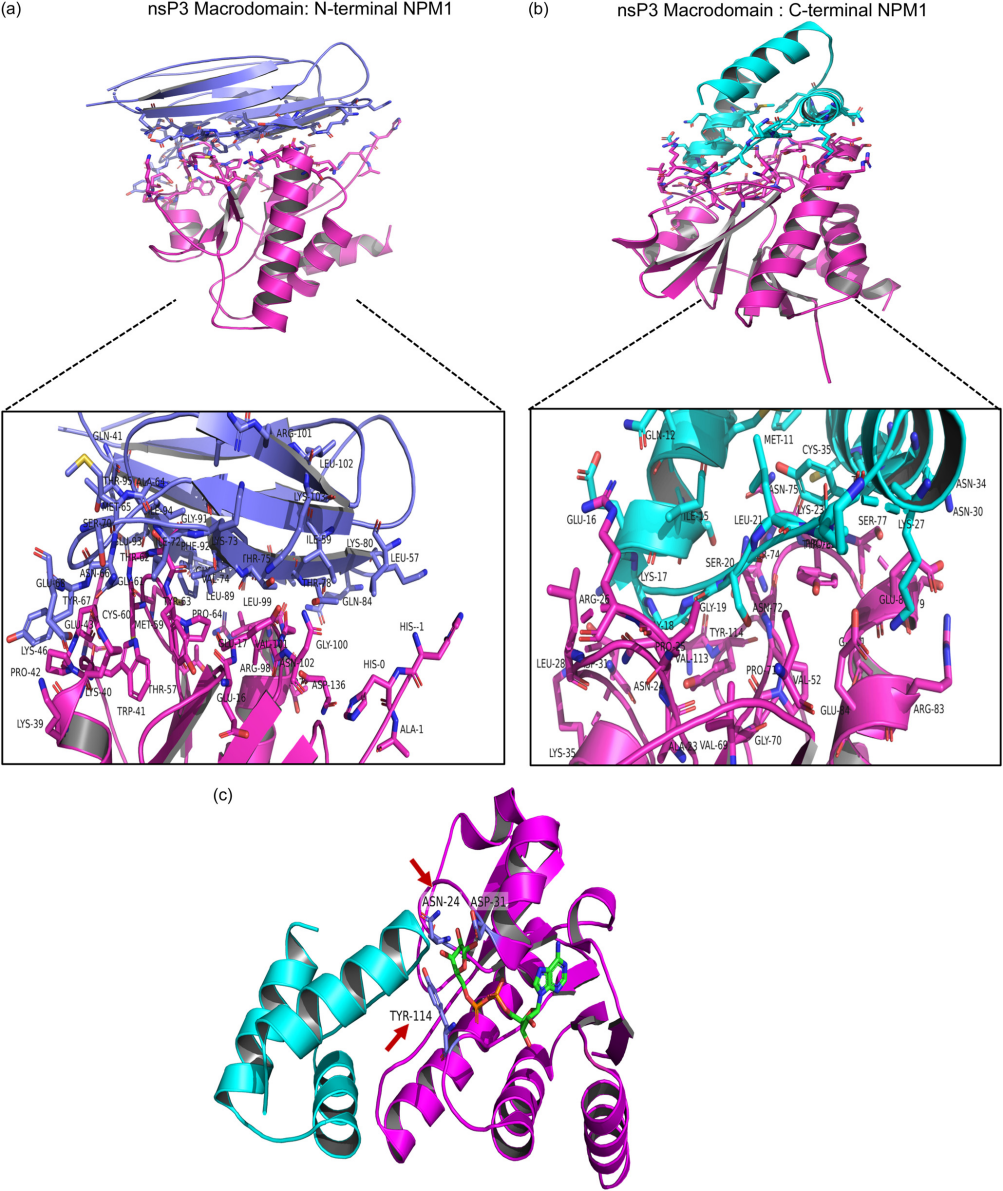
*In silico* analysis predicts that the NPM1 interaction with the CHIKV nsP3 macrodomain is mediated by ADP-ribose-binding residues. The top-ranked complex model from the Cluspro analysis is shown. (a) nsP3 macrodomain N-terminal NPM1 interaction. (b) nsP3 macrodomain C-terminal NPM1 interaction. The NPM1 N terminus is shown in blue, and the NPM1 C terminus is shown in cyan. The macrodomain is shown in pink, and the interacting residues are shown in the stick model. (c) Key residues of the C-terminal NPM1 that interact with the nsP3 macrodomain and are involved in ADP-ribose binding. The residues reported to be involved in alphavirus virulence are indicated by arrows.

Docking analysis was performed to determine the region of NPM1 that interacts with the CHIKV nsP3 macrodomain using the reported three-dimensional (3D) crystal structures of the NPM1 C-terminal domain, NPM1 N-terminal core domain, and CHIKV nsP3 macrodomain in complex with ADP-ribose. In the analysis, the weightage scores for the CHIKV nsP3 macrodomain-NPM1 N-terminal complex and the macrodomain-NPM1 C-terminal complex were −543.7 and −431.9, respectively. Amino acid residues Ala1, Ala2, Glu16, Glu17, Lys39, Lys40, Trp41, Pro42, Glu43, Lys46, Thr57, Cys60, Gly61, Thr62, Tyr63, Pro64, Thr97, Arg98, Leu99, Gly100, Val101, Asn102, and Asp136 of the CHIKV nsP3 macrodomain formed surface contacts with the NPM1 N-terminal core domain to mediate a firm and stable contact (Fig. 11a). Amino acid residues Ala23, Asn24, Pro25, Arg26, Leu28, Asp31, Lys35, Val52, Val69, Gly70, Pro71, Asn72, Ser74, Asn75, Tyrr76, Ser77, Ser79, Glu80, Gly81, Arg83, Glu84, Val113, and Tyr114, Asn24, Asp31, and Tyr114 of the macrodomain were found to be involved in the primary interaction with the NPM1 C-terminal region (Fig. 11b). NPM1 C-terminal interaction with the macrodomain involved Asn24, Asp31, and Tyr114 residues that are close to the C-terminal tail base, which are key residues important for the ADP-ribose binding function of the macrodomain (Fig. 11c).

### NPM1 binds ADP-ribose-binding residues Asn24 and Tyr114 of the CHIKV nsP3 macrodomain.

**FIG 12:**
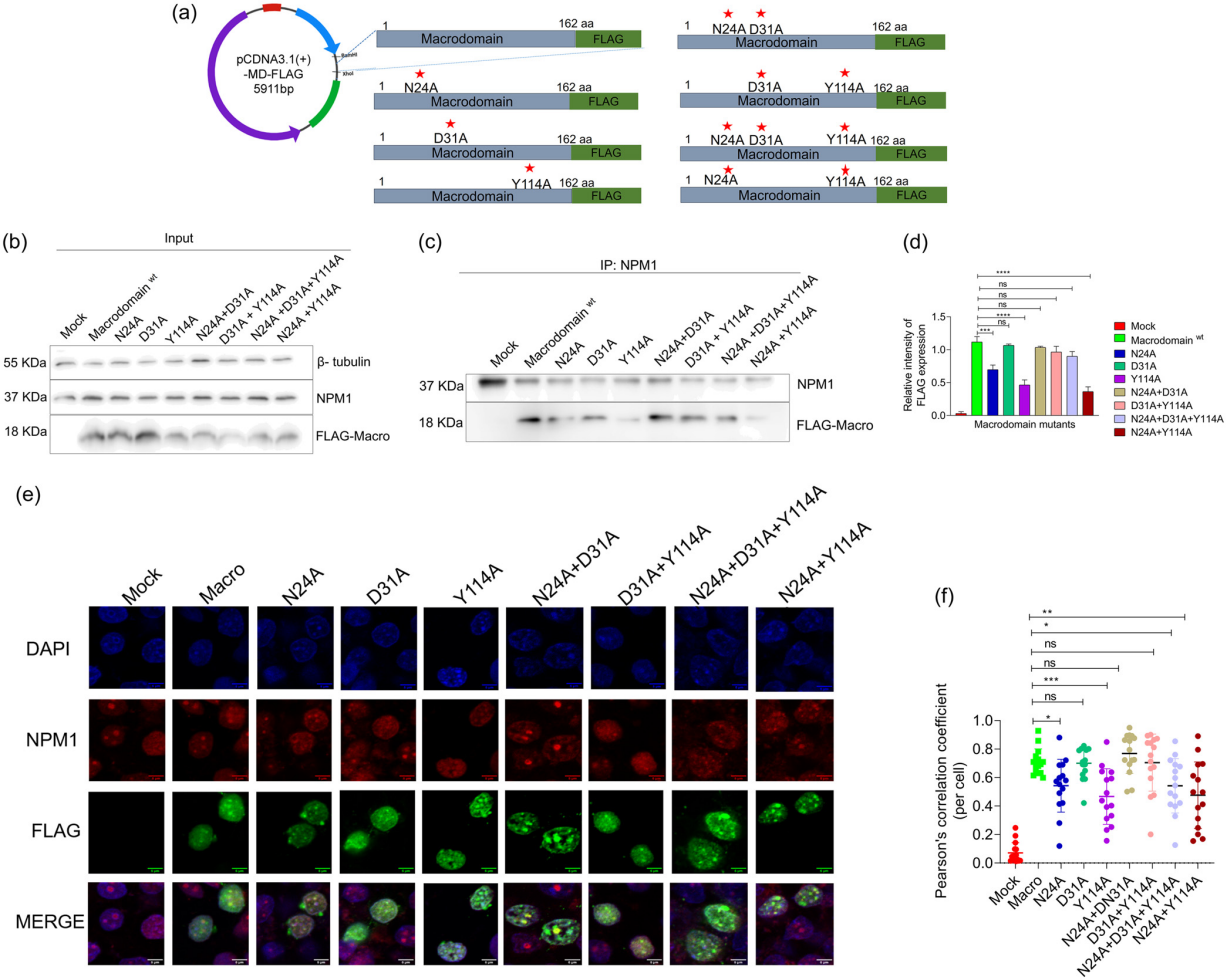
NPM1 binds ADP-ribose binding residues Asn24 and Tyr114 of the CHIKV nsP3 macrodomain. (a) Schematic representation of the generated CHIKV nsP3 macrodomain mutant constructs. (b) HEK293T cells were transfected with pCDNA.1 CHIKV nsP3 wild-type macrodomain or pCDNA3.1 CHIKV nsP3 macrodomain mutants (N24A alone, D31A alone, and Y114A alone single mutants and combination mutants N24A + D31A, D31A + Y114A, N24A + D31A + Y114A, and N24A + Y114A), and the input lysates were immunoblotted for FLAG expression. The expression of NPM1 and β-tubulin is also shown. (c) NPM1 pulldown assays were performed on these lysates, and the immunoprecipitates were probed with anti-FLAG and anti-NPM1 antibodies. (d) Densitometry of the expression levels of FLAG-macrodomain mutants in IP analysis. (e) HEK293T cells were mock transfected or transfected with pCDNA.1 CHIKV nsP3 wild-type macrodomain and mutant constructs and immunostained for NPM1 and FLAG expression 48 h posttransfection. Representative images of three independent experiments are shown; scale bar, 5 μm. (f) Scatter dot plot of co-localization of NPM1 with nsP3 macrodomain mutants. Pearson correlation coefficients in each group was calculated from 15 selected cells. ***, *P* < 0.0003; **, *P* < 0.002; *, *P* < 0.05. Data were analyzed by ordinary one-way ANOVA.

The ADP-ribose-binding residues of the nsP3 macrodomain N24, D31, and Y114 (Fig. 12a) were mutated to alanine by site-directed mutagenesis. The FLAG-tagged mutant proteins were coimmunoprecipitated from transiently transfected HEK293T cells using anti-NPM1 antibody, and the immunoprecipitates were subjected to Western blotting with anti-FLAG antibody to determine the residues critical for NPM1 interaction. There was significant reduction in the interaction of single mutants, N24A and Y114A, as well as the double mutant N24A-Y114A with NPM1, whereas the D31A single mutant showed the same level of interaction as the wild-type protein with NPM1 (Fig. 12b to d). The results confirmed that N24 and Y114 are critical ADP-ribose-binding residues needed for NPM1-CHIKV nsP3 macrodomain interaction.

To further confirm the role of these mutations in their interaction with endogenous NPM1, colocalization studies were done in HEK293T cells transfected with mutant nsP3 macrodomain-expressing plasmids. Immunostaining with anti-NPM1 and anti-FLAG antibodies 48 h posttransfection showed a significantly weak colocalization of NPM1 with N24A/Y114A single mutants and with the combined mutant compared to that of the D31A mutant as well as the wild-type macrodomain protein (Fig. 12e and f).

## DISCUSSION

Several cellular proteins are involved in supporting virus replication or conversely in augmenting the cellular antiviral defense; and these host cellular responses are critical in the outcome of infection (30, 31). Viperin (32), HSP-90 (33), interferons (IFNs) (34), and tetherin (35) are a few such host proteins previously identified to modulate CHIKV infection. We had earlier reported the antiviral role of another host protein, NPM1, against CHIKV (18). In the present study, initial functional experiments using knockdown and overexpression approaches reconfirmed the pronounced antiviral effect of NPM1 on CHIKV. While significant increases in viral replication levels, including viral dsRNA foci, viral proteins, and infectious particles, were observed following NPM1 knockdown (Fig. 1 and 2), the effect was reversed after NPM1 overexpression (Fig. 3). In the present study, we took the lead further forward and elucidated the possible mechanisms on the CHIKV inhibitory effect of NPM1.

The cellular innate immune system, through the autocrine and paracrine actions of type I (IFN-α/β), type II (IFN-γ), and type III (IFN-λ) IFNs, protects cells against viral infections by the induction of ISGs. Since NPM1 has been identified as a transcriptional regulator of ISGs and has been linked to the control of the type II IFN signaling pathway (36–38), we presumed that one of the possible mechanisms of anti-CHIKV activity of NPM1 could be mediated through its effect on ISGs. In the IFN response pathway, IRF-1 is a key transcription factor that induces the expression of ISGs (39). Additionally, IRF-1 has been shown to directly regulate antiviral genes in a type I and type II IFN-independent manner (39–41). Many ISGs are also directly activated by dsRNA or other viral products in an IFN-independent manner, and these are known as viral stress-inducible genes (VSIGs) (42–44). Hence, although we used the U-87 MG cell line in our experiments, which cannot produce endogenous type I IFNs due to defective genes (45), there are possibilities of ISG induction by these cells that permit NPM1 action through their modulation. Earlier studies have shown that ISGs, such as *IRF7* (46), *IFIT1* (47), and *OAS3* (48), play antiviral roles against chikungunya virus. Knockdown of NPM1 reduced the CHIKV infection-triggered induction of the transcripts of these genes, whereas overexpression of NPM1 upregulated their expression (Fig. 4 and 5). We also found that in CHIKV-infected cells, the expression of IRF-1, the key transcription factor in ISG induction (39), is influenced by NPM1 in a time-dependent manner (Fig. 4 and 5). These results demonstrated that NPM1 could modulate the induction of antiviral ISGs and mediate its anti-CHIKV effect.

Positive-sense RNA viruses replicate in the cytoplasm, whereas NPM1 is primarily a nucleolar protein, although it is known to shuttle between both the cytoplasmic and nuclear compartments (22, 23). It was therefore essential to understand where and how NPM1 interacts with CHIKV components to exert the antiviral effect. Our data from infection kinetics of NPM1 localization and analysis of subcellular fractions showed that NPM1 levels in the cytoplasm increase in response to CHIKV infection and indicated that nuclear NPM1 was partially translocated to the cytoplasm (Fig. 6), where it might be interacting with CHIKV replication. Our previous study had shown that cytoplasmic aggregation of NPM1 is essential for its antiviral function, as an oligomerization inhibitor alleviated the antiviral activity (18). Alphaviruses form cytoplasmic vacuoles (CPVs) modified from endosomal and lysosomal membranes along with viral nonstructural proteins, dsRNA, and essential host factors (49), and these virus replication complexes (vRCs) are sites of viral RNA replication. Our colocalization studies indicated that NPM1 colocalizes with viral RNA, thus, possibly forming part of the vRC complex (Fig. 7; Fig. S3 in the supplemental material), which, thereby, forms a site for mediation of the antiviral effect.

To understand how the NPM1-CHIKV interaction happens, we performed an analysis of immunoprecipitated NPM1-binding protein complexes. We identified viral nsP3 as one of the major viral proteins in the complex based on peptide abundance (Fig. 8) along with a number of other host proteins involved in various cellular processes (data not shown). nsP3 is one of the viral proteins in vRCs of alphaviruses, including CHIKV (50). Specific analysis by forward and reciprocal immunoprecipitation assays showed a strong interaction between NPM1 and nsP3, further confirming the presence of NPM1 also in the vRC (Fig. 8).

It has been shown that the NPM1 C-terminal domain interacts with membrane lipids (51) and generates lysate granules rich in ATPase, helicase, and ribonucleoprotein (RNP) components (52). As CHIKV replicates in the cytoplasm, its nsP3 protein is detected in cytoplasmic and perinuclear areas (53–55). Several host proteins are reported to interact with alphavirus nsP3 (11), and proteins such as FHL1, G3BP, SH3KBP1, NAP1L4, BIN1, and CD2AP interact with CHIKV nsP3 via its hypervariable domain (11, 12, 56). It could be possible that an interaction between NPM1 and CHIKV nsP3 may enable RNP rearrangements, attracting cellular mRNA repression/decay machinery that may inhibit viral replication (51, 52). In another possibility, it is known that silencing NPM1 inhibits autophagy (57), and host cells could use NPM1 modulation to inhibit viral replication by modulating autophagy, which is already known to play a role in CHIKV infection (58–60). A third interesting and important possibility emerged from our analysis of the nsP3 protein domains that interact with NPM1. We found that among the three functional domains of nsP3, the macrodomain strongly binds to NPM1 (Fig. 9).

Macrodomains that are present in several viruses (61, 62) alter protein ADP ribosylation, a posttranslational modification wherein ADP-ribose is covalently attached to target proteins by poly-ADP-ribose polymerases (PARPs) using NAD^+^ as a substrate (63, 64). ADP ribosylation of the target protein may change its function, interactions with other proteins, and stability (63, 64), thereby controlling various cellular processes, including DNA repair, transcription, and chromatin remodeling and stress responses (63). ADP ribosylation by PARPs imparts antiviral properties, and the viral macrodomain may counter this innate immune response by removing ADP-ribose groups, thereby interfering in stress granule formation and PARP-mediated antiviral defenses (61, 62). The CHIKV nsP3 macrodomain has ADP-ribose binding and hydrolase activity that are necessary for initiation of virus replication and amplification of the replication complex, respectively, and it results in hydrolysis of ADP-ribose groups from MARylated proteins (9, 10). Mutations in the CHIKV nsP3 macrodomain affect CHIKV virulence in animal models (10, 65).

NPM1 is known to be PARylated by PARP-1 (66) and PARP-2 (67) and to be MARylated by PARP-3 (68), PARP-10, PARP-11 (69), and PARP-14 (70). In line with these observations, in macrodomain pulldown assays and NPM1 coimmunoprecipitation assays followed by probing for pan-ADP-ribose binding, it was confirmed that NPM1 is ADP ribosylated in uninfected and CHIKV-infected cells (Fig. 10). Our results on interaction studies predicted high-affinity binding of both N and C termini of NPM1 to the nsP3 macrodomain, although slightly more for the N terminus, and it may indicate that NPM1 could be another target for the ADP-ribosyl hydrolase activity of the CHIKV nsP3 macrodomain (Fig. 10 and 11). The C-terminal residues of NPM1 were predicted to interact with three residues of the CHIKV nsP3 macrodomain, N24, D31, and Y114 (Fig. 11), which make hydrogen bonds with the OH group on the distal ribose (71), wherein the N24 coordinates the distal ribose and Y114 stabilizes it (9, 62). Interestingly, N24 and Y114 are key amino acid residues of the macrodomain that are known to affect CHIKV virulence in mouse models (10). In earlier studies, targeting the CHIKV nsP3 macrodomain is proposed as a promising antiviral strategy against CHIKV (71, 72). Site-directed mutagenesis and further coimmunoprecipitation and colocalization studies confirmed that NPM1 binds the critical residues N24 and Y114 of the CHIKV nsP3 macrodomain (Fig. 12).

Our results reveal the cross-talk between the ADP-ribosylated NPM1 and the CHIKV nsP3 macrodomain as a critical factor in CHIKV restriction by NPM1. In infected cells, this might be countered by the ADP-ribose hydrolase activity of the virus macrodomain. This would, in turn, help the virus in its perpetual struggle to sustain the intracellular replication countering cellular defenses, including ISG modulation. Overall, our results point to the multiple roles played by NPM1 in CHIKV restriction, making it a promising target for a host-directed approach in antiviral development against chikungunya.

## MATERIALS AND METHODS

### Cell lines and virus.

U-87 MG (a human astrocytic cell line [HTB-14]) cells, Vero cells, and HEK293T cells were purchased from ATCC and were maintained in Dulbecco’s modified Eagle’s medium (DMEM) supplemented with 10% heat-inactivated fetal bovine serum (FBS) and 1× antibiotic-antimycotic mixture (all from Invitrogen). Characterization of the CHIKV viral strain (Rajiv Gandhi Centre for Biotechnology (RGCB) 355/KL08; GenBank accession number GQ428214) used in this study has been previously reported (73). The virus was isolated by three serial passages in Vero cells, and viral titer was quantified by plaque assay on Vero cells before use for infecting U-87 MG cells. All infection experiments were conducted following the Institutional Biosafety Committee (IBSC) guidelines for RGCB.

### Antibodies and reagents.

Rabbit anti-CHIKV E2 polyclonal antibody was prepared in-house, and rabbit anti-CHIKV nsP3 antibody was kindly provided by Andres Merits (University of Tartu, Estonia). The following antibodies were used: mouse anti-NPM1 (Sigma, B0556), rabbit NPM1 (Sigma, HPA011384), mouse monoclonal antibody J2 (dsRNA; SCICONS, J2-1103), normal mouse IgG polyclonal antibody (Sigma, 12-371), Chrome pure rabbit IgG (Jackson Immunoresearch, 011-000-003), mouse monoclonal anti-β-actin (Sigma, A5316), rabbit anti-FLAG antibody (Sigma, F7425), mouse monoclonal anti-β-tubulin (Sigma, T4026), rabbit monoclonal IRF-1 (Cell Signaling Technology, 8478), mouse monoclonal lamin A/C (Cell Signaling Technology, 4777), rabbit monoclonal anti-vinculin (Cell Signaling Technology, 13901), anti-lamin B1 (ab65986), rabbit polyclonal anti-GFP (ab6556), rabbit monoclonal anti-GAPDH (Cell Signaling Technology, 2118), and pan-ADP-ribose-binding agent (MABE1016). Horseradish peroxidase-conjugated antibodies were purchased from Sigma. Donkey anti-mouse IgG H&L (Alexa Fluor 488, Abcam, ab150105), goat anti-rabbit IgG H&L (Alexa Fluor 488, Abcam, ab150077), anti-mouse IgG H&L (Alexa Fluor 647, Jackson Immunoresearch, 115-605-003), and anti-rabbit IgG H&L (Alexa Fluor 647, Jackson Immunoresearch, 111-605-003) were also used. AF1521 macrodomain covalent resin (2506) and negative-control resin (2507) were obtained from Tulip Biolabs (PA, USA).

### siRNA-mediated silencing.

Human NPM1 siRNA SMART pool (M-015737-01-0005), on-TARGET plus nontargeting pool (D-001810-10-05, Dharmacon), NM 001037738 (Sigma, SASI_Hs02_00308362), and siRNA universal negative control (Sigma, SIC001) were transfected into U-87 MG cells using Lipofectamine RNAiMAX (Invitrogen, 13778075) following the manufacturer’s protocol. For knockdown experiments, U-87 MG cells were seeded in six-well plates and grown overnight to a confluence of 70%. All siRNAs were transfected at a final medium concentration of 25 nM using 2.5 μL of Lipofectamine reagent. Twenty-four hours posttransfection, cells were infected with CHIKV at an MOI of 1. NPM1 knockdown was validated by Western blotting; supernatants collected at different time points postinfection were used for plaque assays, and lysates were collected for Western blotting experiments.

### Plaque assay.

Confluent monolayers of Vero cells seeded in 24-well plates were infected with 10-fold dilutions of culture supernatants in DMEM containing 2% FBS, and the virus was allowed to adsorb on cells for 2 h in a 37°C incubator. Cells were then given a phosphate-buffered saline (PBS) wash, and 1 mL of overlay medium (2× DMEM, 2% FBS, and 3% carboxymethylcellulose) was added and incubated for 48 h. Subsequently, the cells were fixed in 30% formalin and stained with 0.05% crystal violet.

### Plasmids.

Plasmid pCDNA3.1-CHIKV nsP3-FLAG, which carries the full-length CHIKV nsP3 gene, was constructed by cloning a NotI-digested PCR fragment into a NotI-digested pCDNA3.1 vector backbone. The three other deletion constructs of CHIKV nsP3 (pCDNA3.1 macro-FLAG [amino acids 1 to 162], pCDNA3.1-AUD-FLAG [amino acids 162 to 318], and pCDNA3.1-HVD-FLAG [amino acids 318 to 530]) were made by cloning the corresponding PCR fragments into the vector pCDNA3.1. NPM1 plasmid constructs pEGFP-NPM WT (17578), nuclear export signal-deleted mutant pEGFP-NPM-NESD (13283), and nuclear localization signal-deleted mutant pEGFP-NPM-NLS1/2D (13287) were obtained from Addgene (USA).

### *In vitro* transcription.

Plasmids pCDNA3.1 CHIKV nsP3 and pCDNA3.1 CHIKV E2 clones were linearized at the C-terminal ApaI site, followed by purification and *in vitro* transcription using an mMESSAGE mMACHINE kit (Ambion, Life Technologies). One microgram of linearized, purified DNA was incubated with 3 μL of T7 polymerase, 10 μL of 2× NTP/CAP, 2 μL of GTP, 2 μL of 10× reaction buffer, and nuclease-free water up to 20 μL for 2 h at 37°C. The yield and integrity of synthesized RNA were analyzed in agarose gels, and the RNA was purified using phenol:chloroform extraction.

### Binding and entry assay.

For binding assays, control siRNA- or NPM1 siRNA-transfected U-87 MG cells were infected with CHIKV at an MOI of 1 and incubated at 4°C on ice for 1 h to allow the virus only to bind, but not enter, cells. For entry assays, incubation was performed at 37°C after infection. Culture supernatants were collected to determine the amount of remaining infectious virions (extracellular) by plaque assay on Vero cells. After PBS washes, cell lysates were collected in TRIzol to quantify intracellular viral RNA by RT-qPCR of the E2 gene.

### Kinetics of infection by RT-qPCR assay.

Control siRNA-treated and NPM1 siRNA-treated cells were infected with CHIKV at an MOI of 1. At the indicated time points postinfection, cells were washed once with PBS and collected in TRIzol (TaKaRa) for subsequent RNA extraction according to the manufacturer’s instructions. RNA samples were reverse transcribed using AMV RT (Promega) and CHIKV E2/nsP3 reverse primers according to the manufacturer’s instructions. Diluted cDNA was used for SYBR-based qPCR (TaKaRa), and obtained threshold cycle (*C_T_*) values were plotted against the log dilutions of the RNA (E2 or nsP3) generated by *in vitro* transcription for the construction of the standard curve. The copy number in the test samples was calculated from the intersection points on the standard curve. Gene-specific primers amplifying a 148-bp region of the CHIKV nsP3-coding sequence and a 133-bp region of the CHIKV E2-coding sequence were used with the following PCR program: 95°C for 30 s; 40× (95°C for 5 s and 60°C for 30 s); and a dissociation curve of 95°C for 15 s, 60 °C for 1 min, and 95°C for 15 s, as predefined. Significance was calculated by two-tailed Student’s *t* test or one-way analysis of variance (ANOVA). Primer sequences are available upon request.

### RT-qPCR.

Control siRNA- or NPM1 siRNA-treated cells were used for knockdown experiments, whereas mock-transfected and GFP-NPM1-transfected cells were used for overexpression experiments. In both cases, at 24 h posttransfection, cells were infected with CHIKV at an MOI of 1. At the indicated time points postinfection, cells were washed once with PBS and collected in TRIzol (TaKaRa) for subsequent RNA extraction according to the manufacturer’s instructions. Total RNA isolation followed by cDNA synthesis and SYBR Green-based RT-qPCR were performed, as previously described (17), using gene-specific primers (Table S1 in the supplemental material). Data were normalized by the level of *ACTB* expression in each sample. The 2^−ΔΔ^*^CT^* method was used to calculate relative expression changes. All experiments were done in three biological and three technical replicates, and the results were analyzed statistically using GraphPad Prism 7 software.

### Western blotting.

U-87 MG cells were either treated with control siRNA or NPM1 siRNA followed by virus infection or mock infection. Cells and cell supernatants were harvested at different time points and pelleted by centrifugation at 5,000 × *g* and 4°C for 5 min. The supernatant was stored at −80°C for plaque assays. The cell pellet was washed with ice-cold PBS and recentrifuged, and cells were lysed with radioimmunoprecipitation assay (RIPA) buffer (Sigma), followed by protein concentration estimation by Bradford assay. Lysates (30 to 50 μg of total protein per lane) were subjected to SDS-PAGE and blotted onto polyvinylidene difluoride (PVDF) membranes (GE Amersham). The membrane was blocked with 5% bovine serum albumin (BSA) or skim milk, followed by subsequent washing and overnight incubation with respective primary antibodies (NPM1, Sigma, B0556; in-house anti-CHIKV E2 rabbit polyclonal serum and anti-rabbit CHIKV nsP3) at 4°C. The next day, after washing the membrane at room temperature for 1 h, the secondary antibody was added and incubated. Membranes were further processed for detection with chemiluminescence (ECL prime reagent GE) and imaging with Versadoc (Bio-Rad) or development on an X-ray sheet. The specific protein intensities were normalized to that of β-actin.

### Transient transfection.

U-87 MG cells were seeded in 6-well plates to obtain 70 to 80% confluence on the day of transfection. Cells were transfected with 4 μg of plasmid pEGFP-NPM WT (Addgene plasmid 17578) using Lipofectamine 2000 (Invitrogen) according to the manufacturer’s instructions. At 6 h posttransfection, the transfection mix was replaced with complete fresh medium (DMEM with 10% FBS). After 24 h, cells were infected with CHIKV at an MOI of 1. The culture supernatants were harvested 48 h after infection, and the virus was titrated on Vero cells by plaque assay. Lysates were subjected to immunoblotting for CHIKV nsP3, CHIKV E2, GFP-NPM1, and endogenous NPM1 expression.

### Confocal fluorescence microscopy.

U-87 MG cells grown on glass coverslips were treated with NPM1 siRNA or control siRNA and infected with CHIKV at an MOI of 1 for 48 h. The cells were fixed with 4% paraformaldehyde in PBS and permeabilized using 0.2% Triton X-100 for 10 min at room temperature, washed with PBS, and blocked by incubating in PBS containing 3% BSA for 1 h at 37°C. After washing with PBS, cells were incubated with antibodies (in-house rabbit anti-CHIKV polyclonal serum against recombinant E2/NPM1/CHIKV nsP3/J2 anti-dsRNA antibody) overnight at 4°C. The next day, cells were incubated with appropriate fluorescently labeled secondary antibodies (Sigma). Nuclear morphology was revealed by 4′,6-diamidino-2-phenylindole (DAPI) staining (final concentration of 1 μg/mL). Coverslips were mounted on glass slides, and fluorescence was observed using an inverted fluorescence microscope.

To examine the colocalization of endogenous NPM1 with CHIKV nsP3-truncated plasmid constructs, HEK293T cells were mock transfected or transfected with pCDNA3.1-CHIKV nsP3 full-length or pCDNA3.1-MD-FLAG, pCDNA3.1-AUD-FLAG, or pCDNA3.1-HVD-FLAG constructs and kept for a 48-h incubation. Cells were washed with PBS once, fixed with 4% paraformaldehyde, permeabilized with 0.2% Triton X-100, and incubated with primary antibodies diluted in 1× PBS containing 3% BSA. The endogenous NPM1 or FLAG-tagged CHIKV nsP3 truncated constructs were detected with mouse anti-NPM1 (1:2,000) or rabbit anti-FLAG (1:400; Sigma), respectively. After washing with PBS, cells were incubated with respective secondary antibodies, and the nuclei were stained with DAPI.

### Microscopy and image analysis.

Immunolabeled samples were imaged with a Nikon A1R, and analysis was performed using Fiji image processing software. To determine cytoplasmic:nuclear protein ratios, whole-cell and nuclear fluorescence intensity of the indicated proteins were first obtained. The nuclear intensity was then subtracted from the whole-cell intensity to get the cytoplasmic protein level, and the data were expressed as cytoplasmic:nuclear fluorescence ratios.

### Nuclear and cytoplasmic fractionation.

Subcellular fractionation was performed according to the Thermo NE-PER nuclear and cytoplasmic extraction kit. Cells were lysed in ice-cold cytoplasmic extraction buffer 1 (CER I), according to the manufacturer’s instructions, vortexed in the tube vigorously, and incubated on ice for 10 min, followed by the addition of cytoplasmic extraction buffer II (CER II). Cells were pelleted for 5 min at 16,000 × *g*. Cytoplasmic fractions were collected separately, and the nuclear pellet was resuspended in nuclear extraction buffer, followed by centrifugation at 16,000 × *g* for 10 min. Nuclear fractions were collected, and lysates were analyzed by Western blotting. Lamin A/C or lamin B1 and β-tubulin were used as controls to check the purity of the fractionation.

### Coimmunoprecipitation.

For coimmunoprecipitation experiments, total cell lysates were prepared in 1 mL of buffer A (10 mM Tris [pH 7.4], 100 mM NaCl, 0.5 mM EDTA, 0.5% NP-40, 1% Triton X-100, and protease inhibitor cocktail [Thermo Fischer]) per 2 × 10^5^ cells and subjected to gentle sonication. Lysates were centrifuged at 12,000 × *g*. The supernatant was incubated with 4 μg of the anti-NPM1 antibody or normal mouse control IgG (IR) overnight at 4°C with constant mixing, followed by incubation at 4°C for 2 h with protein A-Sepharose. Beads were washed extensively, resuspended in 2× SDS-PAGE loading buffer, boiled for 5 min, and used for SDS-PAGE analysis. The selected gel fragment was processed for further LC-MS/MS analysis.

### Reciprocal immunoprecipitation.

HEK293T cells were transfected for reverse immunoprecipitation experiments with 15 μg of pCDNA3.1-CHIKV nsP3 FLAG construct. After 48 h posttransfection, total cell lysates were prepared in 1 mL of buffer A (10 mM Tris [pH 7.4], 100 mM NaCl, 0.5 mM EDTA, 0.5% NP-40, 1% Triton X-100, and protease inhibitor cocktail [Thermo Fischer]) and gently sonicated (1 mL of lysis buffer per 2 × 10^5^ cells was used for lysis). Lysates were centrifuged at 12,000 × *g*. The supernatant was incubated with 4 μg of anti-FLAG antibody or normal rabbit control IgG (IR) overnight at 4°C with mixing, followed by incubation at 4°C for 2 h with protein A-Sepharose. Beads were washed extensively, resuspended in 2× SDS-PAGE loading buffer, boiled for 5 min, and used for Western blotting.

### Protein identification by LC-MS/MS.

NPM1 immunoprecipitation was performed as described previously. SDS-PAGE separated the lysates, and the bands were digested. The generated peptides were analyzed using an LC-MS/MS nanoACQUITY ultraperformance liquid chromatography (UPLC) chromatographic system (Waters, Manchester, UK) and a Synapt G2 High-Definition MS system (HDMSE System; Waters). Liquid chromatography separations were performed on a trap column Symmetry (180 μm × 20 mm C_18_ 5 μm, Waters) and analytical column (75 μm × 200 mm HSS T3 C_18_ 1.8 μm, Waters) at 35°C. The mobile phase consisted of solvent A (0.1% formic acid in water) and solvent B (0.1% formic acid in acetonitrile). A linear gradient with a flow rate of 300 nL/min was applied in the following manner: B 1% (0 min), 1% (3 min), 40% (43 min), 80% (46 min), 80% (50 min), 1% (51 min), and 1% (51 min). The mass spectrometer was interfaced with an electrospray ion source and operated in positive mode. The flow of the Ion-mobility spectrometry (IMS) nitrogen gas was set to 90 mL/min, while the electrospray capillary voltage was optimized to 3,400 V. A positive false-discovery rate (FDR) of 1 for the identification of peptides and proteins and unique peptide counts of ≥1 were the criteria used to identify proteins. Carbamidomethylation at cysteine residues was set as a variable modification, and methionine oxidation was set as a fixed modification. The data were collected and analyzed using Progenesis QI for Proteomics V4.2 (Non-Linear Dynamics, Waters) software.

### Mass spectrometry data analysis.

The acquired MS^E^ spectra were analyzed using Progenesis QI for Proteomics V4.2 (Non-Linear Dynamics, Waters) software for protein identification and quantification. Data processing included lock mass correction postacquisition. Processing parameters for Progenesis were as follows: noise reduction thresholds for low-energy scan ion, 150 counts; high-energy scan ion, 30 counts. The protein identifications were acquired by searching against the human and chikungunya viral database downloaded from UniProt. The parameters for protein identification were made in such a way that a peptide was required to have at least one fragment ion match, a protein was required to have at least three fragment ion matches, and a protein was required to have at least one peptide match for identification. Cysteine carbamidomethylation was chosen as a variable modification, and oxidation of methionine was chosen as a fixed modification. An FDR of ≤1.0% was used during the database search. Trypsin was selected as the enzyme used with a specificity of one missed cleavage. Data sets were evaluated using the Hi-N algorithm, which resolves peptide conflicts and uses the average intensity of the three most abundant unique peptides for a protein. After peptide and protein identification, the abundance of each peptide was calculated from all its constituent peptide ions. For each protein, the abundances of the *n* most abundant peptides were averaged to provide a reading for the protein signal. In Progenesis QI for proteomics, the ranking of peptide abundance is based on the integrated value across all the runs allowed by the lack of missing values and accurate alignment. This gives added confidence in the peptide selection, taking all runs into account to make the ranking robust. This averaged reading allows relative quantification of the same protein across runs and further filtering and processing of the data using an in-house developed script in R and R studio (version 2021.09.2), and corresponding data are visualized using the R “ggplot2” package.

### Macrodomain pulldown assay.

To examine whether NPM1 is a target for ADP ribosylation, we performed pulldown assays using AF1521 macrodomain covalent resin (Tulip Biolabs, PA, USA) along with a negative-control resin. For pulldown assays, total cell lysates from mock- and CHIKV-infected cell lysates were prepared in 500 μL of RIPA buffer containing protease inhibitor cocktail and were subjected to gentle sonication. Lysates were centrifuged at 12,000 × *g*, and around 0.2 mg of clarified lysate solubilized in RIPA buffer was added to a microcentrifuge tube containing 25 μL of washed AF1521 and negative-control resin. The extract-resin mixture was incubated on a rotator for 4 h at 4°C to allow binding of target proteins to the resin. Beads were washed extensively, resuspended in 2× SDS-PAGE loading buffer, boiled for 5 min, and used for Western blotting to detect ADP-ribosylated NPM1.

### NPM1 pulldown assay.

Mock- and CHIKV-infected cell lysates were prepared in 1 mL of buffer A (10 mM Tris [pH 7.4], 100 mM NaCl, 0.5 mM EDTA, 0.5% NP-40, 1% Triton X-100, and protease inhibitor cocktail [Thermo Fischer]) per 2 × 10^5^ cells, subjected to gentle sonication, and centrifuged at 12,000 × *g*. Lysates were treated with 100 ng of poly-ADP-ribose glycohydrolase (PARG; Sigma, SRP8023) and were subjected to immunoprecipitation using anti-NPM1 antibody. The immunoprecipitated proteins were probed with anti-pan-ADP-ribose binding agent to detect ADP ribosylation.

### *In silico* prediction of NPM1 and CHIKV nsP3 macrodomain interaction.

The 3D crystal structures of the NPM1 C-terminal domain (2VXD), NPM1 N-terminal core (2P1B), and macrodomain of CHIKV nsP3 (3GPG) were downloaded from Protein Data Bank. To find the largest cluster that would represent the likely models of the complex, an exhaustive search was conducted using an algorithm implemented in ClusPro for protein-protein docking (74). ClusPro 2.0 uses the fast fourier transform (FFT) correlation approach, root mean square deviation (RMSD)-based clustering of the generated structures, and refinement of selected structures that reveal the binding mode of the chikungunya virus macrodomain with the N-terminal core domain and C-terminal domain of NPM1. Based on the top-scoring function, the predicted top models were chosen, and PyMol was used to identify and visualize the residues involved in the interaction.

### Site-directed mutagenesis.

Site-directed mutagenesis was performed in the pCDNA3.1-CHIKV macrodomain-FLAG plasmid backbone using a QuickChange site-directed mutagenesis kit (Stratagene, CA). All the selected amino acids were mutated to alanine, and a total of seven mutant constructs were generated. Primer details are given in Table S1.

### Statistical analysis.

Statistical analyses were performed using GraphPad Prism 7.01 software, and a *P* value of <0.05 was considered statistically significant. Graphical representation and analyses were done using RStudio 2021.09.2.
