# Supplementary material for: Host Factor Nucleophosmin 1 (NPM1/B23) Exerts Antiviral Effects against Chikungunya Virus by Its Interaction with Viral Nonstructural Protein 3
Source: Microbiol Spectr. 2023 Jul 6;11(4):e05371-22. doi: 10.1128/spectrum.05371-22 (PMC10433958; doi:10.1128/spectrum.05371-22)
Supplement: Supplemental file 1 — Supplemental material. Download spectrum.05371-22-s0001.docx, DOCX file, 1.4 MB [file spectrum.05371-22-s0001.docx]

**Host factor Nucleophosmin 1 (NPM1/B23) exerts antiviral effect against Chikungunya virus by its interaction with viral non-structural protein 3**

Parvanendhu Pradeep^a,b^, Krishnankutty Chandrika Sivakumar^c^, Easwaran Sreekumar^a,d^*

^a^ Molecular Virology Laboratory,

Rajiv Gandhi Centre for Biotechnology (RGCB),

Thiruvananthapuram, Kerala, INDIA- 695014

^b^ Research Centre, University of Kerala,

Thiruvananthapuram-695034, Kerala, India

^c^ Bioinformatics facility, Rajiv Gandhi Centre for Biotechnology (RGCB),

Thiruvananthapuram, Kerala, 695014, India.

^d^ Institute of Advanced Virology (IAV), Bio 360 Life Science Park, Thonnakkal, Thiruvananthapuram 695317, Kerala, India

**Supplementary Data**

**
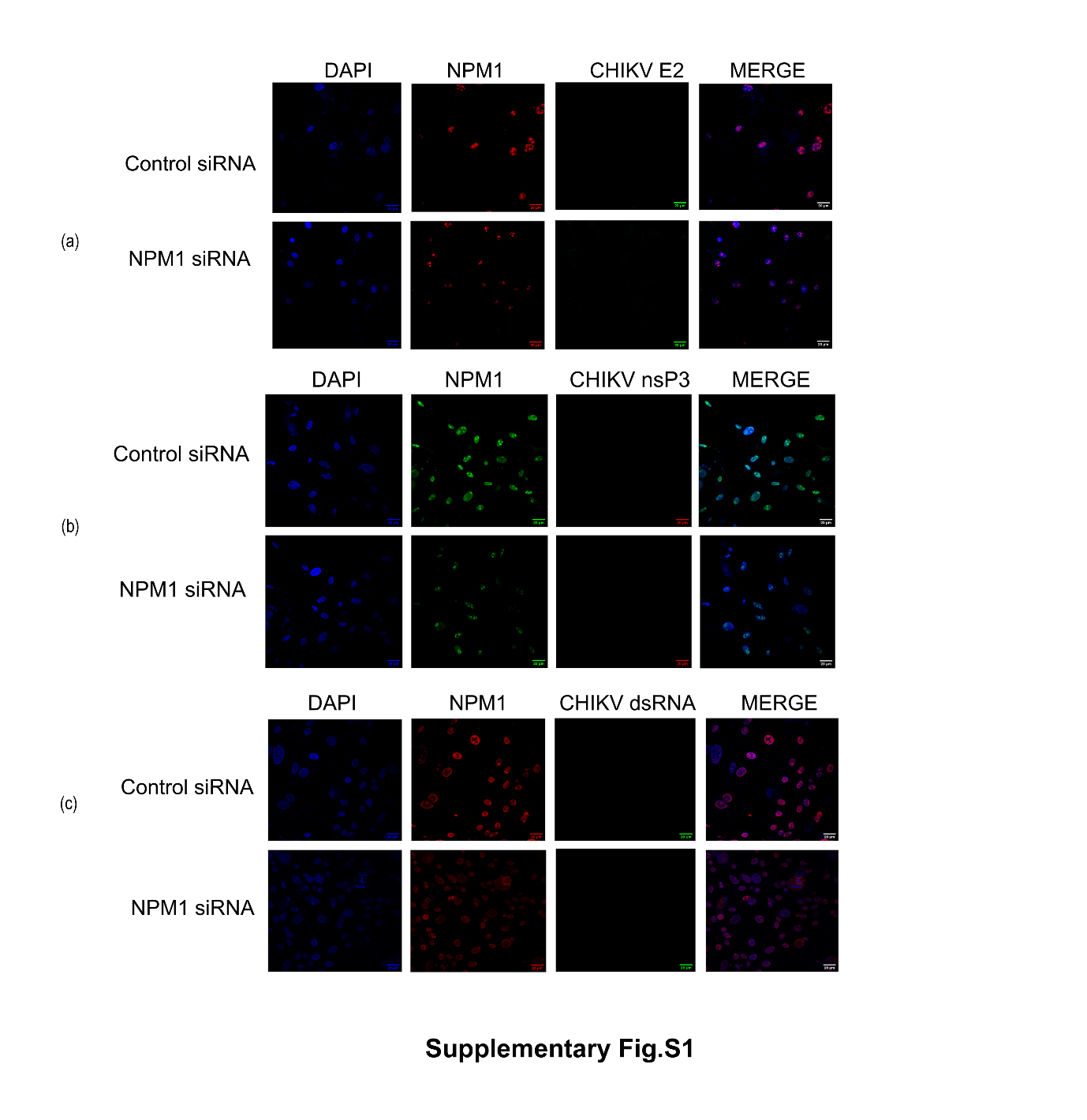
**

**Fig. S1:** **Knockdown of NPM1 expression enhances CHIKV replication.**

(a)Immunofluorescence was performed on U-87 MG cells transfected with NPM1 siRNA1 or control siRNA and 24 hours post transfection mock infected and stained for NPM1 and CHIKV E2 expression at 24hpi.(b) Experiment performed as in (a) and stained for NPM1 and CHIKV nsP3 expression. (c)Immunofluorescence was performed on U-87 MG cells transfected with NPM1 siRNA1 or control siRNA and stained for NPM1 and dsRNA foci in control siRNA or NPM1 siRNA1 treated cells. (Scale bar: 20µm).

**
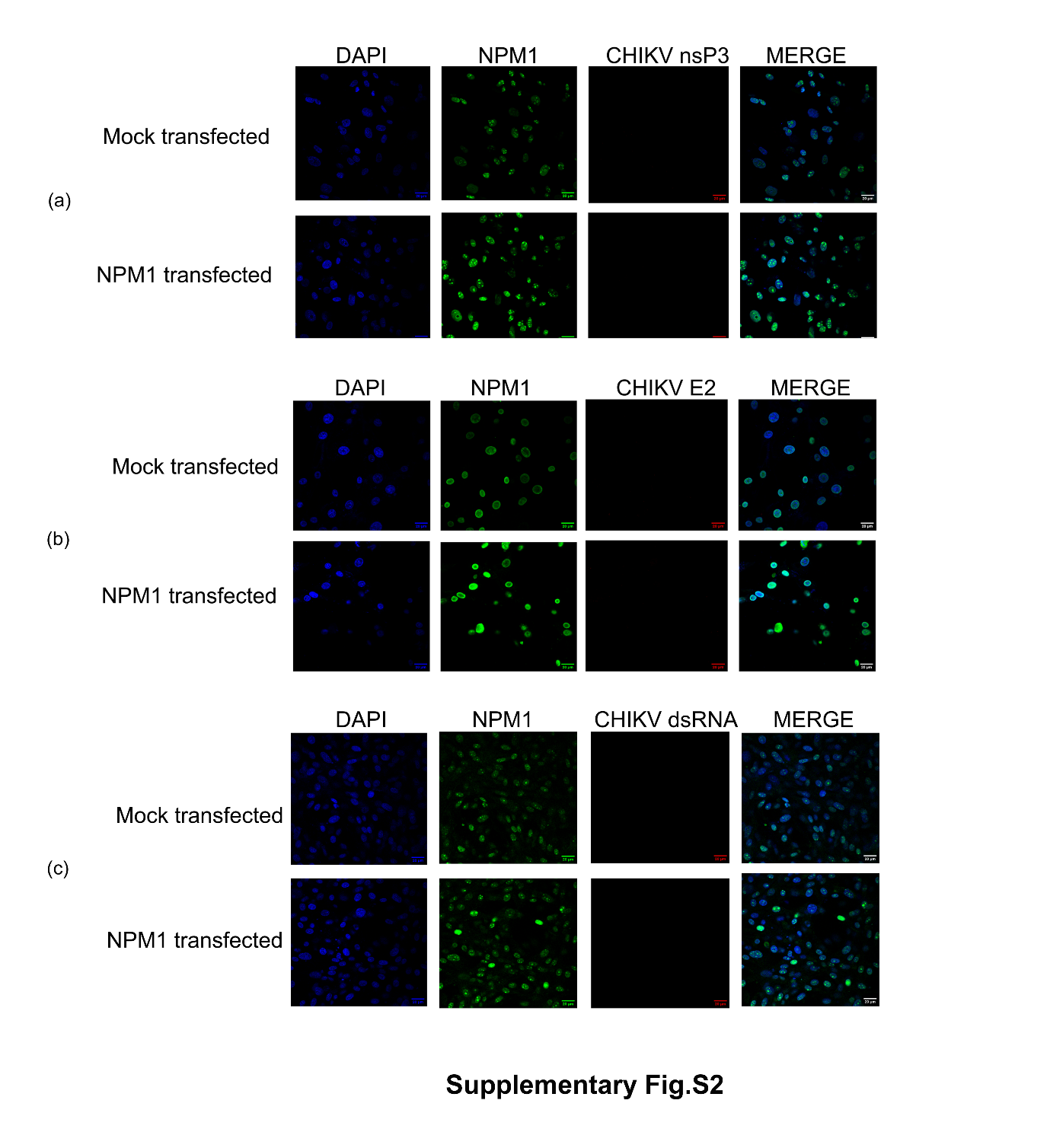
**

**Fig. S2:** **NPM1 overexpression suppresses CHIKV replication**

Immunofluorescence detection of NPM1 and CHIKV nsP3 expression.(a) U-87 MG cells transfected with NPM1 vector or mock vector and 24 hours post transfection mock infected and stained for CHIKV nsP3 and NPM1 as indicated.(Scale bar: 20µm ). (b)Experiment performed as in (a) and immunostained for NPM1 and CHIKV E2 expression. (c)Experiment was performed as in (a), and stained for dsRNA and quantified . To quantify the dsRNA foci, we choose a total of 15 different fields from three independent experiments (n=3).“***” p-value <0.0005, Un-paired t-test. (Scale bar: 20µm ).

**
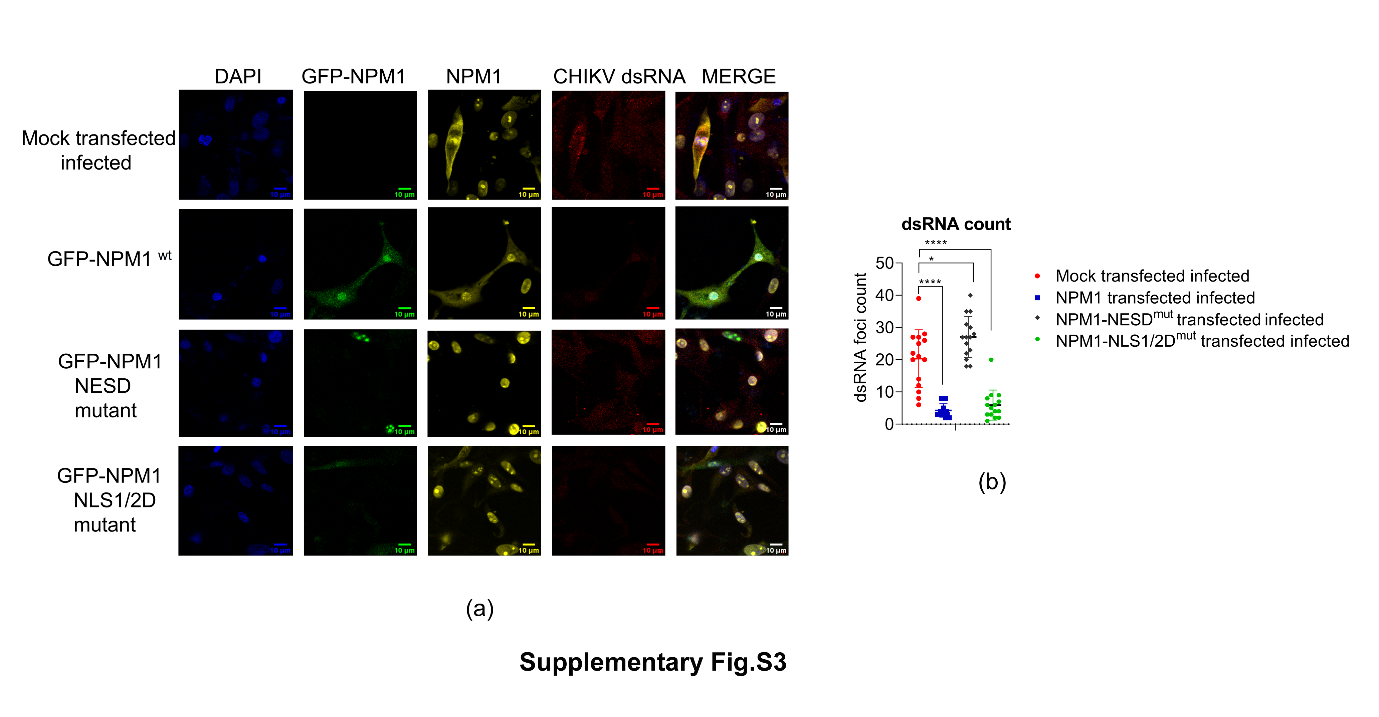
**

**Fig. S3: Cytoplasmic re-localization of NPM1 is important for its anti-CHIKV activity**

1. U-87 MG cells were transfected with mock or NPM1 expression vectors containing either a wild-type construct or NPM1-NESD mutant, which has nuclear export sequence deletion or NPM1-NLS1/2D mutant which has nuclear localization signal deletion. Cells were infected with CHIKV at MOI 1 24h.p.t, and cells were fixed at 48h p.i, stained with an antibody to GFP(GFP-NPM1), endogenous NPM1 and anti-dsRNA, and imaged by confocal microscopy.“****” p-value <0.0001, “**” p-value <0.005, Ordinary one-way ANOVA. Sidak’s multiple comparison test.(Scale bar: 10µm) (b)Quantification of dsRNA foci, a marker of viral genome replication in mock plasmid transfected, NPM1 wild-type(NPM1wt), NPM1-NESD, and NPM1-NLS1/2D plasmid transfected infected cells 48 hours post-infection from 15 different fields from two independent experiments.

**Supplementary Table 1**: Oligonucleotide Primers used in the study.

| **Oligo Name** | **Sequence (5’-3’)** |
| --- | --- |
| ChkEnv2F | TGCCATGAGCACCAAGGACAACTTCAA |
| ChkEnv2FLAGR | TTATCACTTATCGTCGTCATCCTTGTAATCCGCTTTAGCTGTTCTGATG |
| ChknsP3F | TTATCAATGATGGTGATGGTGATGTAACTCGTCCGTGT |
| ChknsP3FLAGR | TTATCACTTATCGTCGTCGTCATCCTTGTAATCATAACTCGTCGTCCGTGT |
| RTCHIKVENV2F | ACAGACGGGACGCTGAAAAT |
| RTCHIKVENV2R | TTACAAATAGCCCCGCCCTC |
| RTCHIKVNSP3F | CATGGCTAGCGTCCGATTCT |
| RTCHIKVNSP3R | GCTTGATGCTCCGAAGGAGA |
| H-IFIT1F | CTGCCTAATTTACAGCAACC |
| H-IFIT1R | TGATCCAAGACTCTGTTTTC |
| H-OAS3F | AGTGTACCAAGATCTCCAAG |
| H-OAS3R | ATGGTCCAGTAGATACAGAG |
| H-IRF1F | CCAAGAGGAAGTCATGTG |
| H-IRF1R | TAGCCTGGAACTGTGTAG |
| H-IRF7F | TGGTCCTGGTGAAGCTGGAA |
| H-IRF7R | GATGTCGTCATAGAGGCTGTTGG |
| H-β-ACTIN F | CCGCAAAGACCTGTACGCCAACAC |
| H-β-ACTIN R | GCTGATCCACATCTGCTGGAAGGT |
| N24A MUT FP | GTCAACGCCGCTGCCCCTCGCGGGTTA |
| N24A MUT RP | TAACCCGCGAGGGCCAGCGGCGTTGAC |
| D31A MUT FP | GGGTTACCGGGTGCCGGTGTTTGCAAG |
| D31A MUT RP | CTTGCAAACACCGCCACCCGGTAACCC |
| Y114A MUT FP | CTCTCCACAGGTGTAGCCTCAGGAGGGAAAGAC |
| Y114A MUT RP | GTCTTTCCCTCCTGAGGCTACACCTGTGGAGAG |
